# Laser-driven growth of structurally defined transition metal oxide nanocrystals on carbon nitride photoelectrodes in milliseconds

**DOI:** 10.1038/s41467-021-23367-7

**Published:** 2021-05-28

**Authors:** Junfang Zhang, Yajun Zou, Stephan Eickelmann, Christian Njel, Tobias Heil, Sebastian Ronneberger, Volker Strauss, Peter H. Seeberger, Aleksandr Savateev, Felix F. Loeffler

**Affiliations:** 1 Max Planck Institute of Colloids and Interfaces, Potsdam, Germany; 2 Department of Chemistry and Biochemistry, Freie Universität Berlin, Berlin, Germany; 3 Institute for Applied Materials (IAM) and Karlsruhe Nano Micro Facility (KNMF), Karlsruhe Institute of Technology (KIT), Eggenstein-Leopoldshafen, Germany

**Keywords:** Electrochemistry, Nanoscale materials, Nanoscale materials

## Abstract

Fabrication of hybrid photoelectrodes on a subsecond timescale with low energy consumption and possessing high photocurrent densities remains a centerpiece for successful implementation of photoelectrocatalytic synthesis of fuels and value-added chemicals. Here, we introduce a laser-driven technology to print sensitizers with desired morphologies and layer thickness onto different substrates, such as glass, carbon, or carbon nitride (CN). The specially designed process uses a thin polymer reactor impregnated with transition metal salts, confining the growth of transition metal oxide (TMO) nanostructures on the interface in milliseconds, while their morphology can be tuned by the laser. Multiple nano-p-n junctions at the interface increase the electron/hole lifetime by efficient charge trapping. A hybrid copper oxide/CN photoanode with optimal architecture reaches 10 times higher photocurrents than the pristine CN photoanode. This technology provides a modular approach to build a library of TMO-based composite films, enabling the creation of materials for diverse applications.

## Abstract (web summary)

Fabrication of hybrid photoelectrodes on a subsecond timescale with low energy consumption remains a challenge. Here, the authors report a modular approach, laser-driven transfer synthesis, to build a library of structurally defined transition metal oxide composite films, enabling the creation of materials for diverse applications.

## Introduction

Conversion of abundant solar energy into fuels and value-added chemicals in photoelectrochemical cells received enormous attention as a technology capable of easing the environmental impact^1^. Successful implementation of this conversion requires durable, inexpensive photoelectrodes enabling high photocurrents and low overpotentials. As a cost-efficient and sustainable semiconductor, polymeric carbon nitride (CN) is a promising material to fulfill such requirements^2,3^. The main challenges of using CNs in photoelectrochemistry are poor electron mobility and fast charge recombination, which have limited broad application^4,5^.

Forming heterojunctions with other materials, such as transition metal oxides (TMOs), is reported as an efficient solution for charge seperation^5–7^. For example, the incorporation of copper oxide (CuO) into the CN matrix induces electron delocalization to increase electron mobility in the composite material^8^. At the same time, CuO with a narrow bandgap (~1.4 eV) can sensitize CN (~2.7 eV bandgap) to harvest larger portions of the solar spectrum^9^. A variety of CuO/CN composites have been applied in different fields, such as anode materials for lithium-ion batteries^10^, photocatalysts for the decomposition of organic pollutants^11^, or voltammetric sensors for the detection of glucose^12^.

Significant efforts have been devoted to the development of synthetic procedures for the preparation of TMO/CN composite materials, including copolymerization^13,14^, photodeposition^15^, microwave-assisted^16^, and hydrothermal methods^17,18^. These processes yield well-defined structures during bulk synthesis of the materials, but they are not suitable for the fabrication of composite films with diverse micro-/nanostructures. Moreover, the most common method, (hydro)thermal treatment, requires a high input of thermal power and long processing times^19^. Even though a physical CN sputtering method has been introduced to generate TMO/CN films with precisely controlled composition, this method shows large interlayer distances with limited interaction and charge transport^20^. The introduction of additional components, such as graphene, aims to improve the performance, nevertheless, results in inefficient systems^21–23^. The above-mentioned issues are responsible for the underperformance of these films in photoelectric semiconductor devices. Therefore, a flexible and modular strategy is required to generate micro-/nano-organized composite films.

We developed the laser-driven transfer synthesis (LTRAS) technology from the laser-induced forward transfer (LIFT) principle. Unlike the traditional (pulsed) laser ablation methods, which usually release the synthesized particles into liquids^24^, LIFT is a versatile maskless method for the transfer of thin-film surface patterns. It is realized in a wide range of variations, for depositing precise and minute amounts of almost any material onto surfaces^25^. For example, metals can be transferred by direct laser melting and ejection, using high-power pulsed lasers^26^. A notable variant using pulsed lasers, called matrix-assisted pulsed laser evaporation direct-write (MAPLE DW)^27^, enables the transfer of thermally and mechanically sensitive materials, such as (nano)particles (e.g., ceramics, alloys, polymers). In addition, also bacteria^28^ or biomolecules^29–31^ can be transferred, by introducing a sacrificial polymer^32^ or laser absorber, enabling more gentle modes of transfer, via ejection, blister (blister-actuated LIFT) and jet formation, or direct contact^33,34^. However, all these existing techniques, including our own previous works, are mainly focused on the transfer of materials instead of in situ synthesis of materials.

Here, we show that our continuous wave laser process cannot only transfer but, at the same time, also drive a chemical reaction to generate precisely controlled materials at the interface of different substrates (e.g., glass, fluorine-doped tin oxide (FTO), carbon, CN). In the laser system, the configuration of the composite film can be flexibly tuned in terms of macroscopic location on the substrate (by the laser irradiation pattern), microscopic particle shape and size (e.g., by laser energy), as well as the types of materials (by different precursor materials or sequential deposition of several precursors). We use the LTRAS method to generate CuO/CN composite films as photoanodes. Thin-layer CuO nanostructures with controllable size and shape are obtained on the CN substrate. Nano-structured p-n junctions are created due to the spatial organization of the composite layers. The CuO morphology significantly influences charge transport and distributions in the films, which further changes the efficiency of photogenerated charge separation and transfer. The photocurrent of the synthetic CuO/CN photoanodes induced under white light is up to ten times higher than the photocurrent of the photoanode made of CN only in 0.1 M NaOH solution. The incident photon-to-electron conversion efficiency (IPCE) values of CuO/CN at 415 and 455 nm reach up to 11.46% and 3.41%, respectively. These composite electrodes are used as non-enzymatic biosensors for glucose under ultra-low operation potential of 0.1 V (vs. Ag/AgCl reference electrode) and irradiation with visible light. Furthermore, the LTRAS method is not limited to Cu-salts—other TMO nanostructures, such as CoO and NiO on the CN film, are readily available via this rapid synthesis procedure.

## Results and discussion

### Establishing the laser-driven transfer and synthesis method

**Fig. 1:**
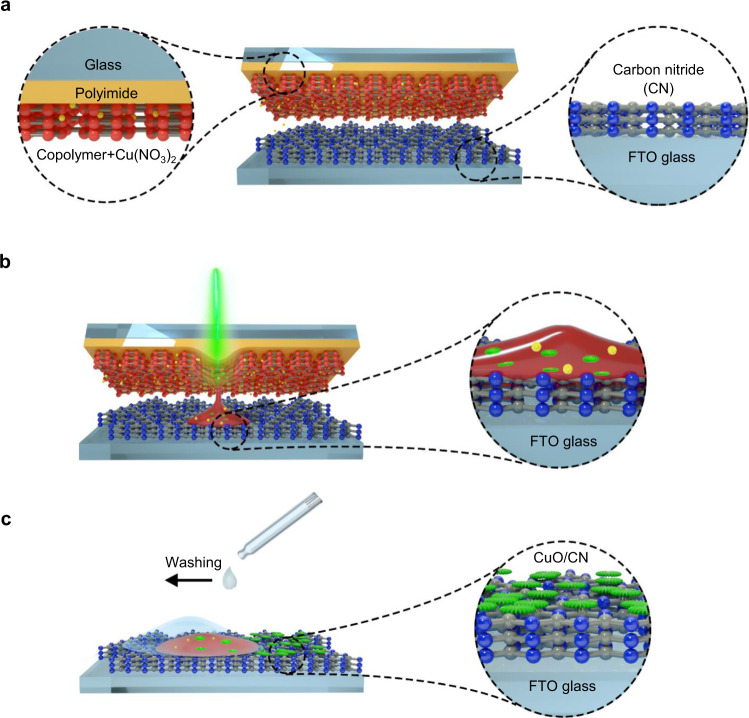
Principle of the laser-driven transfer synthesis (LTRAS) process for the generation of structurally defined transition metal oxide/carbon nitride (TMO/CN) composite films. **a** Laser irradiation transfers material from a donor to an acceptor surface. The donor slide is prepared by spin coating a mixture of dissolved copolymer together with transition metal (TM) precursor onto a polyimide-coated glass slide. The acceptor slide is prepared by vapor deposition polymerization of CN onto a fluorine-doped tin oxide (FTO) glass slide. **b** The laser rapidly heats, melts, and transfers the donor material. At the same time, the decomposition temperature of the metal precursor is reached and TMO structures are formed. **c** After a short rinsing with acetone, the TMO/CN composite film is ready.

LTRAS is based on the photothermic effect: focused laser irradiation generates a confined temperature field at the desired position^35^. Precursor salts are transferred inside the melted polymer and converted into metal oxide nanoparticles within milliseconds (Fig. 1). In general, this process requires a donor and acceptor surface. A homogenous CN film of 300–500 nm thickness was generated on an FTO glass slide by vapor deposition polymerization (VDP)^36,37^ to prepare the “acceptor slide” (Supplementary Fig. 1). A “donor slide” was prepared by spin-coating a solution of metal precursor (first, we used Cu(NO_3_)_2_·3H_2_O) and styrene-acrylic copolymer (S-LEC) onto the surface of a glass slide, bearing a polyimide film (Supplementary Fig. 2). To transfer the metal precursor onto the CN substrate, we placed the donor slide on top of the acceptor slide and irradiated the donor slide with a laser setup (Supplementary Figs. 3 and 4). As the light passes through the donor glass slide it is absorbed by the polyimide layer, which converts light into thermal energy. Thereby, the copolymer with the embedded metal precursor is melted and transferred in a direct contact mechanism to the acceptor slide, as we have reported earlier^34^. This step can be repeated many times with different donor slides, such that the thickness of the transferred layer can be easily adjusted. In addition, the LTRAS process shows successful transfer even on an ultrathin CN film with a thickness of around 50 nm (Supplementary Fig. 5), which proves this method is not limited by the thickness of the CN film on the acceptor slide.

**Fig. 2:**
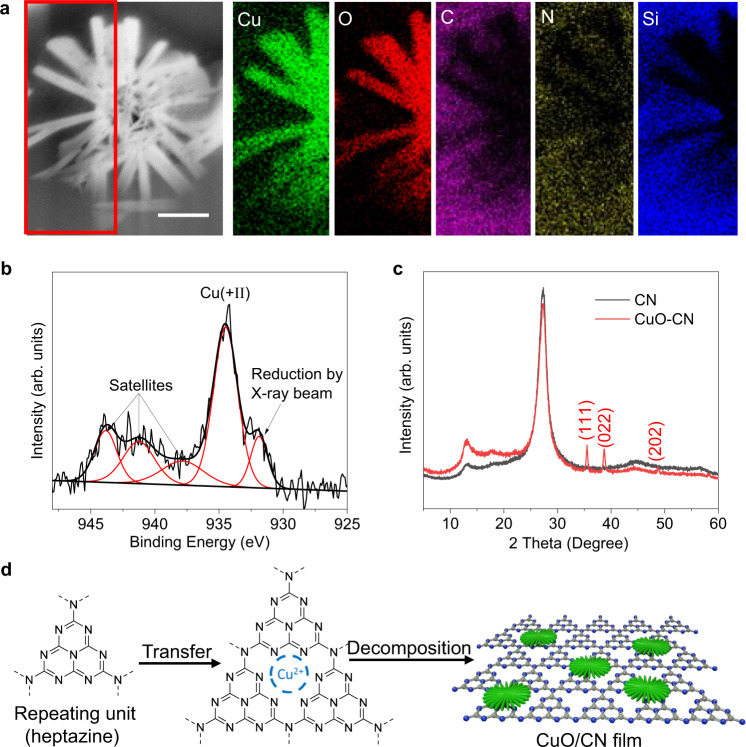
Characterization of the CuO/carbon nitride (CN) composite material generated by LTRAS. **a** SEM image and EDX elemental maps of the transferred composite material (scale bar 2 µm). **b** High-resolution XPS binding energy spectrum of Cu 2*p*. **c** XRD spectrum of CN and CuO/CN composite films. **d** Schematic illustration of the synthetic route during the transfer process.

Scanning electron microscopy (SEM) imaging of the acceptor slide after transfer reveals star-like structures (Fig. 2a). Further analysis using energy-dispersive X-ray spectroscopy (EDX) indicates that these structures consist of copper and oxygen, while carbon and nitrogen are observed only within the CN substrate.

To investigate the oxidation states of copper, X-ray photoelectron spectroscopy (XPS) was performed on the composite films. The full scan spectrum (Supplementary Fig. 6a) shows characteristic peaks of all elements: C, N, Cu, and O from the composite material and Si from the glass substrate, which is consistent with the EDX mapping results. The Cu 2*p* spectrum is normally composed of the 2*p*_3/2_–2*p*_1/2_ doublet, separated by 20 eV with a 2:1 intensity ratio due to spin-orbit coupling. Here, only the Cu 2*p*_3/2_ peaks are considered for the copper chemical states. The peak Cu 2*p*_3/2_ located at 934.5 eV with its shake-up satellites is a signature of various Cu (+II) species, such as CuO and Cu(NO_3_)_2_ (Fig. 2b). Based on the presence of a nitrogen peak around 407 eV, corresponding to the nitrate compound (Supplementary Fig. 6c), the copper detected on the surface of the CuO/CN composite corresponds to a mixture of CuO and Cu(NO_3_)_2_. The small Cu 2*p*_3/2_ peak at the low binding energy corresponds to the reduction product of Cu (+II) under XPS analysis^38^. For the X-ray powder diffraction pattern (XRD, Fig. 2c) of the composite films, the additional peaks at 35.5, 38.6, and 48.7 can be assigned to the (111), (002), and (202) facets in the monoclinic phase of CuO (JCPDS no.48-1548) respectively.

These results indicate that during the LTRAS process, we not only transferred metal precursors onto the CN film but also covered cupric nitrate to defined CuO (Fig. 2d). To further investigate the CuO–CN interaction, we peeled off several CuO/CN films from acceptor slides after laser transfer and put the films into water. We stirred the solution overnight and treated it with ultrasound for 3 h to try to separate the CuO from the CN layer. However, in TEM, we could hardly find single CuO particles, which confirms the high affinity of CuO to CN and proves that the composite is quite stable. This affinity is likely caused by the coordinate bonding of the partially occupied Cu-3d shell and neighboring N regions of the CN^39,40^.

**Fig. 3:**
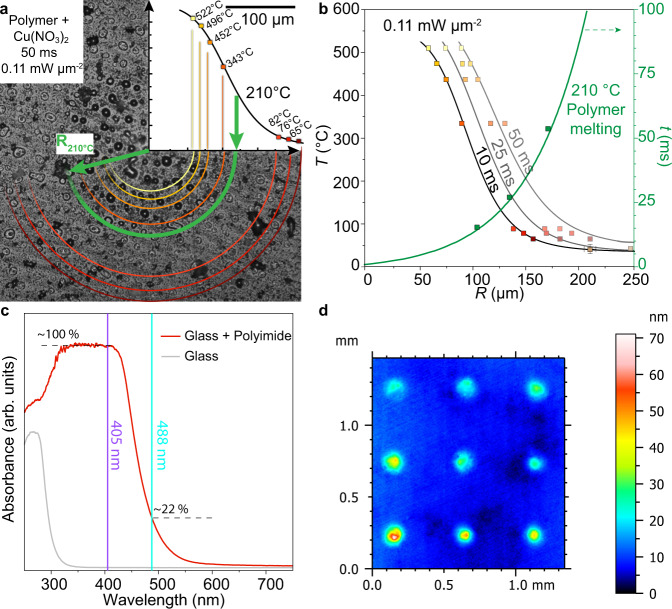
Thermal characterization of the laser process. **a** Thermal map of a spot on a donor slide after 50 ms laser irradiation. Colored rings represent the overlay of isotherms. **b** Thermal profiles for different irradiation times and the radius of melting isotherm (green curve). **c** UV–VIS spectra of glass and polyimide-covered glass. **d** Vertical scanning interferometry (VSI) measurement of laser-transferred material spots in a gradient pattern (effective power density top to bottom 0.107, 0.132, 0.156 mW µm^−2^, irradiation time left to right 40, 30, 20 ms).

Conventionally, the synthesis of CuO is conducted in furnaces at 100–500 °C for 0.5–36 h (Supplementary Table 1), employing either hydrothermal or thermal decomposition methods^41–45^. In contrast, the synthesis proceeds within milliseconds with the LTRAS method (Supplementary Fig. 7). The focused laser irradiation results in high energetic efficiency by rapid heating at the desired position. As we have reported recently^46^, the thermal map of a 50 ms laser irradiation spot (Fig. 3a) on a donor slide features >500 °C in the spot center. The donor film area, which surpasses the melting temperature of the copolymer (210 °C)^46^, is efficiently transferred to the acceptor slide. The melting temperature of S-LEC (210 °C, blue circle) is higher than the decomposition temperature of Cu(NO_3_)_2_ (170 °C), indicating that CuO is the predominant product in the transferred area. The thermal profiles (Fig. 3b) show that longer irradiation times can not only induce higher temperatures but also increase the melting area. Therefore, more material is transferred upon longer irradiation times (see Supplementary Fig. 4 and the following description for a detailed calculation of effective irradiation times).

Besides irradiation time, the synthesis process can also be influenced by the laser wavelength (here 405 or 488 nm), due to higher absorption at shorter wavelengths by the polyimide (Fig. 3c). At 405 nm, the absorption of polyimide is around five times higher than at 488 nm, causing a higher temperature and more material to be transferred. The thickness of the transferred material was measured by vertical scanning interferometry (VSI, Fig. 3d). Gradients with different effective laser power densities (0.107–0.156 mW µm^−2^) and irradiation times (20–40 ms) were generated (Supplementary Fig. 8). In comparison to the diameters of the spots that are around 150 µm, their thickness is in the nanometer regime. Even with the higher laser power density of 0.156 mW µm^−2^ and 40 ms irradiation time, the thickness is still below 60 nm. During the transfer, the melted copolymer serves as a nanoscale reactor for the generation of CuO nanocrystals. The design of this polymer reactor restricts the growth of CuO in the axial direction that preferentially results in confined 2D structures, attached to the CN surface.

### Facile control of nanocrystal morphology

**Fig. 4:**
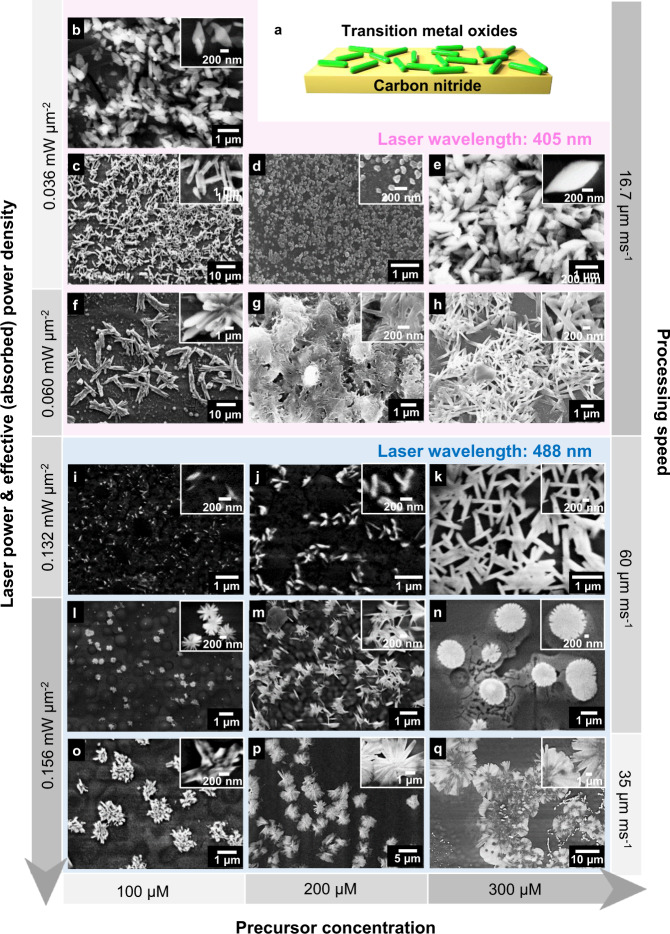
SEM images of transition metal oxide (TMO) structures synthesized by LTRAS and their corresponding synthesis parameters. **a** Structure of TMO/CN composite films. **b**, **e**, **h**–**q** CuO/CN. **c**, **f** NiO/CN. **d**, **g** CoO/CN.

Rod-like, star-like, and rhombic structures were obtained via LTRAS with sizes ranging from ~200 nm to ~5 µm (Fig. 4). Length/size control was achieved by changing the concentration of the metal precursor in the polymer mixture of the donor slide. When we decreased the concentration of Cu(NO_3_)_2_ from 300 µM to 100 µM, the size of the CuO rods (Fig. 4i–k) decreased from 450 nm to 200 nm. Moreover, irradiation time and laser power influence the shape of the CuO structures. A fast scanning speed (60 µm ms^−1^) and relatively low laser power density (0.132 mW µm^−2^) form nanorods (Fig. 4i–k), whereas slower scanning (35 µm ms^−1^) or higher power (0.156 mW µm^−2^) cause the formation of 2D star-shaped structures (Fig. 4l, n, p).

Upon heating of the polymer and precursor mixture by the laser (Supplementary Fig. 9), the polymer melts, increasing its solubility. Since slower scanning speed causes longer irradiation time, more precursor is transferred and more CuO nanocrystals are generated. When the laser is switched off, the polymer begins to solidify again. Due to this phase change, the solubility drastically decreases, forcing the growth of larger CuO nanocrystals. The nanocrystals attach to the same facets of the crystallization seeds. Assuming a higher concentration of CuO nanocrystals in the mixture at a slower scanning speed, the probability of faults, i.e., attachment of the nanocrystal to a wrong facet, is higher, thus under such conditions star-like nanostructures are formed. On the other hand, faster-scanning speed, and therefore shorter irradiation time, produces fewer CuO nanocrystals that attach exclusively along the [010] CuO lattice fringe, giving rise to nanorods (Supplementary Fig. 10).

As mentioned before, the laser wavelength can influence the nanostructure shape as well. For a 405 nm laser, significantly less power is required due to the high absorption of polyimide at this wavelength. Here, with an effective laser power density of 0.036 mW µm^−2^ and a speed of 16.7 µm ms^−1^ (about 2–4 times slower than in the 488 nm system), large amounts of material were transferred to form CuO nanorhombi (Fig. 4b, e). In this case, CuO nanostructures even pile up and form multilayers (Fig. 4b), while at 488 nm, all structures are thin-layer. It should be noted that laser power densities above 0.060 mW µm^−2^ at 405 nm destroy the polyimide layer.

LTRAS is not limited to the ultrafast synthesis of CuO nanostructures but was used to synthesize other TMOs, such as NiO (Fig. 4c, f) and CoO (Fig. 4d, g). After laser transfer, the materials on the acceptor slide were characterized by XRD (Supplementary Fig. 11) and EDX mapping (Supplementary Fig. 12). Rod-like NiO was obtained from Ni(NO_3_)_2_ at 405 nm laser irradiation (Fig. 4c). When we increased the effective laser power density from 0.036 mW µm^−2^ to 0.060 mW µm^−2^, these NiO rods formed star-like nanostructures (Fig. 4f). In the case of cobalt, laser irradiation converts cobalt nitrate into oxide nanostructures of defined shapes. The CoO nanospheres observed by SEM are around 160 nm in diameter (Fig. 4d). Higher power induces a completely different shape of planar CoO assemblies (Fig. 4g), illustrating the potential of this technology for morphology control.

So far, LTRAS offers a flexible way to grow different TMOs structures on CN films. In fact, this method is also compatible with other substrates, for example, bare glass, FTO glass, and carbon-coated glass (Supplementary Figs. 13–15). In addition, the transferred polymer significantly influences the growth process. We changed the transferred polymer on the donor slides from S-LEC to polyvinylpyrrolidone (PVP), which also offers high reproducibility and accuracy (Supplementary Fig. 16). However, the morphology of the synthesized material is quite different: instead of particles distributed in the transferred polymer phase, a continuous and macroporous layer is formed with the PVP-based donor slides (Supplementary Fig. 17). This allows for an additional degree of flexibility in the LTRAS method, enabling the synthesis of different structures.

### Composite photoanodes by LTRAS

**Fig. 5:**
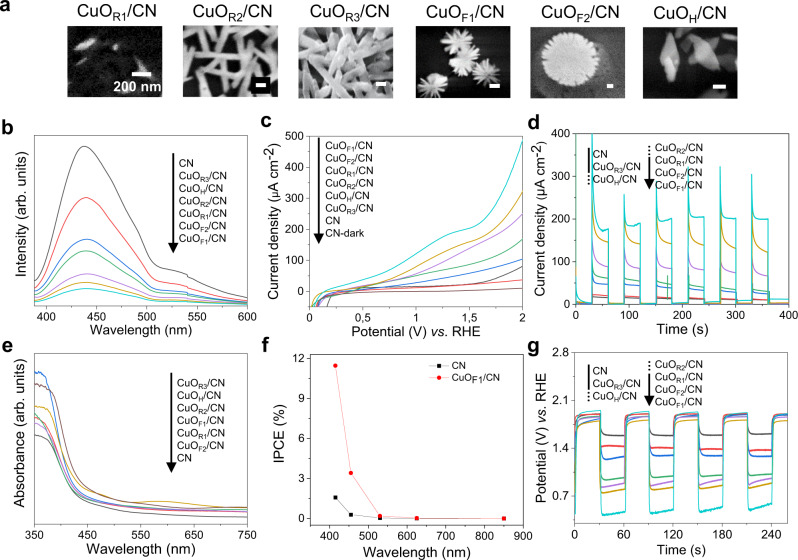
Photoelectric performance of carbon nitride (CN) and composite electrodes. **a** Different structures of composite films and their corresponding name. **b** Photoluminescence spectra. **c** Linear sweep voltammetry curves under white light. **d** Transient photocurrent response of CN-based electrodes in 0.1 M NaOH solution at 1.23 V vs. RHE (reversible hydrogen electrode) under white light. **e** UV–visible absorption spectra. **f** Incident photon-to-electron conversion efficiency (IPCE) plots of CN and CuO_F1_/CN electrodes as a function of incident photon wavelength by applying a potential of 1.23 V vs. RHE in 0.1 M NaOH, and (**g**) chronopotentiometry under white light.

The photoluminescence (PL) measurements of pure CN and CuO/CN composite photoelectrodes at room temperature and upon excitation at 365 nm (Fig. 5b) reveal a significant quenching of the PL intensity after deposition of CuO onto the electrodes. This indicates the presence of new deactivation paths, such as charge separation between CN and CuO. As the only composite electrode with a multi-layer CuO structure, CuO_R3_/CN gave a higher PL intensity than the other structures. Thus, charge migration and separation ability in multi-layer structures seem to be limited.

In addition, the shape and size of CuO nanostructures also influence the performance of the as-synthesized electrodes. Photocurrents of different electrodes under linearly increasing operation potential were measured in 0.1 M NaOH solution (Fig. 5c). All composite electrodes show higher photocurrent under white light irradiation compared to the pristine CN electrode, especially the electrodes with thin-layer CuO structures, which is a result of higher accessibility of the CN surface to incident photons (Fig. 5d). Star-shaped CuO with a smaller size (CuO_F1_/CN) exhibits the largest photocurrent density (172 µA cm^−2^ at 1.23 V vs. reversible hydrogen electrode (RHE)), which is about nine times higher than the pristine CN electrode and comparable or higher than several other works (Supplementary Table 2). It has been observed that crystal orientations show different reactivity^47^. Therefore, the overall reactivity can be influenced by the particle shape^48^. Star-like CuO/CN electrodes yield higher current densities in comparison to those with rod-like structures. This might be caused by the difference in crystal orientation in these two structures. When we add a known hole scavenger (triethanolamine) to the electrolyte, the current density of CuO_F1_/CN can be further increased to 225 µA cm^−2^ at 1.23 V vs. RHE (Supplementary Fig. 18). Besides, CuO_F1_/CN composite electrodes show a lower onset potential (0.14 V vs. RHE) in comparison to pristine CN electrodes (0.38 V vs. RHE). The faradaic efficiency of CuO_F1_/CN electrodes for oxygen evolution reaction is found to be 40.3% at 1.23 V vs. RHE (Supplementary Fig. 19).

Overall, the magnitude of the photocurrent correlates well with the results of steady-state fluorescence measurements—the lower fluorescence intensity, the higher the photocurrent (Supplementary Fig. 20). To compare the LTRAS method to other growth strategies of CuO on CN, we followed two other strategies to synthesize Cu doped CN: photodeposition (Supplementary Figs. 21–23) and co-polymerization (Supplementary Fig. 25). All as-prepared electrodes yielded much lower photocurrent densities than those prepared with the LTRAS method (Supplementary Figs. 24 and 26).

The LTRAS-synthesized CuO/CN composite electrodes show enhanced absorption and a slight red shift in comparison to the pristine CN electrodes (Fig. 5e). According to the UV–visible spectra, composite electrodes absorb photons with wavelengths <420 nm stronger compared to the bare CN electrode, indicating the synergistic effect of CuO and CN. The absorption at wavelengths >420 nm is mainly caused by the narrow bandgap of CuO. Enhanced absorption in the blue (455 nm) can be observed even though the CuO layer is ultrathin (<100 nm). The IPCE values of CuO_F1_/CN at 415 nm and 455 nm reach up to 11.46% and 3.41%, respectively, which is much higher than pristine CN with only 1.58% at 415 nm and 0.29% at 455 nm (Fig. 5f and Supplementary Fig. 27). The redshift of the IPCE onset wavelength suggests that the CuO functions as an absorber in the composite electrodes.

The potential of the photoelectrodes under constant current (50 µA) shows a prompt response to light irradiation and shifts to more negative regions in all cases, suggesting a decrease of electrode resistance (Fig. 5g). The composite electrode with the star-shaped structures CuO_F1_ gives the largest drop of resistance under light irradiation. Even under blue light irradiation, the potential of the CuO_F1_/CN electrode can be lowered to 0.1–0.2 V vs. Ag/AgCl, to achieve the same current as for the pristine CN electrode at 0.9 V (Supplementary Fig. 28). Therefore, this electrode can be applied for low-power sensing with advanced performance. For instance, we used a CuO_F1_/CN electrode as a photoanode for the detection of glucose. In general, 0.3–0.7 V vs. Ag/AgCl operation potential is necessary to register the response of an electrode to glucose (Supplementary Table 3). However, in our case, 0.1 V can be sufficient for the CuO_F1_/CN photoelectrode according to the results of chronopotentiometry, while the remaining energy demand is supplied via irradiation with light (Supplementary Fig. 29).

### Formation of micro p-n junctions in polymer reactor

The significantly enhanced photoelectric properties are induced by the special design of the composite structure upon addition of CuO. By plotting (*αhν*)^2^ as a function of *hν* (Supplementary Fig. 30a), the band gaps of the CN and CuO were calculated to be 2.74 eV and 1.41 eV respectively, which are in accordance with the onset of absorption. Since the flat band potential can be calculated by the intercept of the tangent in the Mott Schottky (MS) plots with the *X*-axis, we could estimate the valence band edge of CuO and conduction band edge of CN with a potential difference of around 0.1 eV in respect to the flat band potential (Supplementary Fig. 30b, c)^49^. Composite CuO_F1_/CN electrodes exhibit an inverted V-shape MS plot (Supplementary Fig. 30d), suggesting both the n- and p-type semiconductors are in contact with the electrolyte. More importantly, this inverted V-shape MS plot confirms the presence of p-n junctions^49^.

**Fig. 6:**
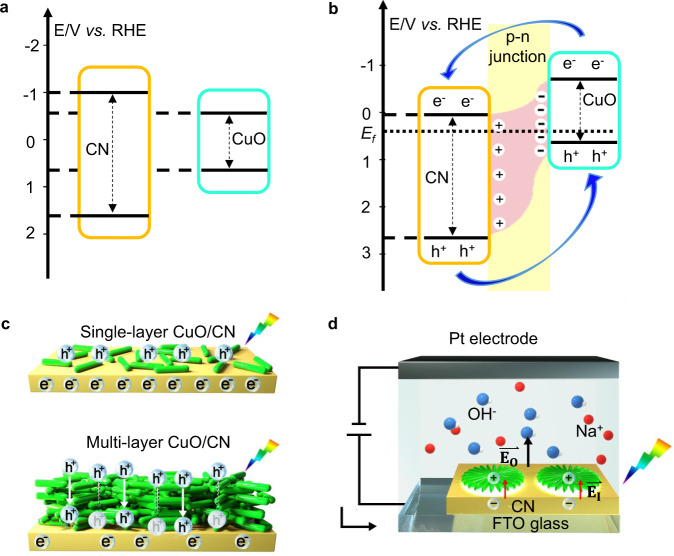
Energy diagrams of the materials and charge transport properties. **a** Energy diagram of CN (carbon nitride) and CuO. **b** Energy diagram of the CuO/CN heterostructure, when CuO and carbon nitride are in direct contact. **c** Transport of photogenerated charges in thin-layer and multi-layer CuO/CN electrodes. **d** Star-shaped CuO on CN functions as a photoanode under light irradiation and applied potential. RHE reversible hydrogen electrode, *E*_*f*_ energy of Fermi-level, $\vec{{{\bf{E}}}_{{\bf{I}}}}$ internal electric filed, $\vec{{{\bf{E}}}_{{\bf{O}}}}$ applied electric field.

Based on these results, a band structure is proposed for CN, CuO, and the composite CuO_F1_/CN electrodes (Fig. 6a, b). Due to the Fermi level alignment, the band edge of CN shifts downwards, while that of CuO shifts upwards^49,50^. Therefore, the conduction band of CN is more positive in comparison to CuO, which leads to the transfer of photogenerated electrons to the CN, and holes to the CuO. Another factor influencing the charge transfer pathway is the p-n junction. The diffusion and recombination of charge carriers at the interface induce an internal electric field in the depletion region^51^. This electric field forces electrons to move from the p-type (CuO) to the n-type (CN) semiconductor. Hence, the photogenerated electrons and holes in the composite CuO_F1_/CN electrodes are spatially isolated, which leads to efficient charge separation.

In contrast, in composite films with multi-layer CuO structures (such as Cu_OR3_/CN, Fig. 5a), the photogenerated charges on the outer surface of the CuO suffer from long-distance diffusion (Fig. 6c). Due to the poor conductivity of CuO, fast recombination occurs before these charge carriers reach the interface between CuO and CN (Supplementary Fig. 31). In addition, CuO has a high absorption coefficient (e.g., 1.2·10^5^ cm^−1^ at 420 nm)^51^ in the visible range (Supplementary Fig. 32). Applying the Lambert–Beer law, we estimate that a 100 nm thick layer of CuO is sufficient to absorb 92% of the incident light, i.e., only 8% of incident light reaches the CN layer. In the case of the thick multi-layer CuO, it efficiently blocks the CN layer located underneath from light. This explains why composite electrodes with a thinner CuO layer have significantly better photoelectric properties than thicker CuO structures on the CN. When the composite structures are applied as photoanodes (Fig. 6d), the capacitance of the space-charge region is decreased, resulting in a more efficient flow of charges^7,52^. Therefore, under light irradiation and applied bias potential, micro-scale p-n junctions formed in the composite material can strongly improve the charge separation and reduce charge transfer resistance.

Concluding, we introduce the LTRAS method for simultaneous transfer of transition metal precursors and controlled in situ synthesis of defined TMO structures within milliseconds on different substrates, such as glass, FTO, carbon, and CN. For CuO, this method allows control over the size and shape of CuO nanostructures by careful selection of the laser parameters. The 2D CuO assemblies on the surface of CN form micro-scale p-n junctions with an oriented internal electric field. More importantly, thin-layer CuO can improve the efficiency of charge diffusion and increase the number of photoactive sites in the CN underneath, such that as-prepared composite electrodes possess significantly enhanced photoelectric properties. These composite films can be used for different applications such as low-voltage biosensors for glucose. The photoanode made by deposition of CuO_F1_/CN on an FTO substrate shows sensitive current responses upon successive injection of glucose even under 3–6 times lower operation potential than other works. This highly flexible, time-, and energy-efficient LTRAS method can be applied to the synthesis of other TMOs, such as NiO and CoO with defined morphologies. This technology provides composite photoanodes with high photocurrent and a facile strategy for the rapid growth of structurally defined TMOs on carbon-based substrates.

## Methods

### Four hundred and eighty-eight-nanometer LTRAS machine setup

We use a 200 mW TOPTICA iBeam smart 488-S laser with a wavelength of 488 nm (TOPTICA Photonics AG, Germany), that can be flexibly tuned in power and pulse duration. The laser is passed through a 1:10 beam expander, before entering a Racoon 11 laser scanning system (ARGES GmbH, Germany), which is equipped with an f-Theta lens (S4LFT5110/322, Sill Optics GmbH, Germany), as shown for LIFT-based array generation (Supplementary Fig. 3)^53^. This setup allows us to scan the laser focus with a defined speed in a plane of 66 mm × 66 mm, enabling reproducible irradiation of a surface at various positions within the lasing area, with a 1/e² laser spot diameter of 18 µm (Supplementary Fig. 4).

### Four hundred and five-nanometer LTRAS machine setup

A second LTRAS setup is equipped with a 405 nm laser with 100 mW as shown in Supplementary Fig. 33. Briefly, a commercial laser engraving system was upgraded with a microcontroller, to have full control over pattern, laser power, and laser pulse duration^31^. The laser is attached to two mechanical axes, which move the laser in a plane above the sample surface. The 1/e² laser spot diameter is 42 µm (Supplementary Fig. 4).

### Thermal map and profiles

We measured the surface temperatures, which occur during the laser transfer of a single spot^46^. Polyimide donor slides were coated with different long alkanes and irradiated, resulting in a defined melting and evaporation pattern of the alkanes. Since the melting and boiling temperatures are known, we could reconstruct the temperature maps for specific lasing parameters.

In the same setup, a donor slide, coated with Cu(NO_3_)_2_ embedded in the copolymer, was irradiated with different lasing parameters. Optical microscopy of these irradiation patterns gives the temperature profiles, corresponding to morphological changes (Fig. 3a) induced by different lasing conditions. The morphological changes can be measured and result in an isothermal plot (Fig. 3b). By comparing these values with our previous mappings, the surface temperature profile can be extrapolated.

### Preparation of donor slides

In total, 10, 20 or 30 mg Cu(NO_3_)_2_·*x*H_2_O (99%, Acros) and 30 mg copolymer (S-LEC PLT 7552, Sekisui Chemical GmbH, Germany) were dissolved in the mixture of 50 µL N, N-dimethylformamide, 100 µL acetonitrile and 350 µL dichloromethane. All solvents were used as obtained from Sigma-Aldrich. We spin-coated the solution on polyimide film (Kapton HN 100 type, Dupont, USA) covered glasses at 80 rps. The thickness of the obtained polymer film was measured to be around 1.5 µm in vertical scanning interferometry (Supplementary Fig. 34). The obtained slides are the so-called “donor slides”. Due to the difference in solubility, 10 mg Ni(NO_3_)_2_.6H_2_O (99%, Roth) and 20 mg Co(NO_3_)_2_.6H_2_O (98%, Sigma-Aldrich) precursors were used in the preparation of the donor slides for the NiO and CoO nanostructures, respectively.

### Vertical scanning interferometer

We used a smartWLI compact (Gesellschaft für Bild- und Signalverarbeitung (GBS) mbH, Illmenau, Germany) with low magnification (5× Nikon CF IC Epi Plan DI—Mirau) to show the thickness maps and profiles in large area. The donor slides were partly washed by isopropanol to show the thickness of polymer films after spin-coating. The acceptor slides after laser irradiation were measured directly without further treatment.

### Preparation of CN electrodes

A clean FTO coated glass (6 cm × 6 cm, 7 Ω/sq, Merck) covered a 29.5 mL rectangular alumina crucible with 5 g melamine (99%, Alfa Aesar). We placed the crucible in a furnace (CMF-1200, Carbolite Gero, UK), set to 550 °C, incrementing by 1.5 °C min^−1^, and kept at 550 °C for 3 h under nitrogen. The as-prepared slides were cut into small pieces (1 × 1.5 cm) and used as CN electrodes.

### Preparation of TMO/CN electrodes

The CN electrodes, as prepared in the previous step, serve as the “acceptor slides” for the laser-assisted generation of TMO/CN electrodes. We placed the donor slides on top of the acceptor slides and loaded them into the sample stage of the laser system. The following laser patterning step was performed either with the 488 nm or 405 nm laser LTRAS machine setup. Laser power and processing speed were controlled by the scanning software (ARGES GmbH, Wackersdorf/Bayern, Germany). We used dichloromethane and/or acetone to wash away the remaining copolymer after laser irradiation and the obtained acceptor slides were used as TMO/CN electrodes.

### Photoelectrochemical measurements

Transient photocurrent density, linear sweep voltammograms, and chronopotentiometry were measured by a three-electrode potentiostat (BioLogic MPG2). A Pt electrode (ALS Co., Ltd, Japan), an Ag/AgCl on sat. KCl filling solution (ALS Co., Ltd., Japan), and 0.1 M NaOH solution are used as the counter electrode, reference electrode, and electrolyte, respectively. The photocurrent with reference to RHE is calculated with the following formula:

1 $${E}_{({RHE})}={E}_{{\mathrm{Ag}}/{\mathrm{AgCl}}}+0.059{pH}+{E}_{{\mathrm{Ag}}/{\mathrm{AgCl}}}^{0}$$

where ${E}_{{\mathrm{Ag}}/{\mathrm{AgCl}}}$ is the applied working potential, ${E}_{{\mathrm{Ag}}/{\mathrm{AgCl}}}^{0}$ = 0.1976 V at 25 °C.

Faradaic efficiency (FE) is measured at 1.23 V vs. RHE and calculated by the following equation:

2 $${FE}\left( \% \right)=\frac{m\cdot n\cdot {{F}}}{I\cdot t}\times 100$$

where *m* is the amount of oxygen (mol), *n* is the number of reaction electrons, *F* is the Faraday constant, *I* is the photocurrent (A), and *t* is the reaction time (s).

Incident photon-to-current conversion efficiency (IPCE) is calculated by the following formula:

3 $${IPCE}\left( \% \right)=\frac{{J}_{{Ph}}({\rm{A}}\cdot {{\rm{cm}}}^{-2})\times 1240({\rm{V}}\cdot {\rm{nm}})}{\lambda ({\rm{nm}})\times {J}_{{Lig}ht}({\rm{W\cdot{{cm}}}}^{-2})}$$

where *J*_*Ph*_ is the photocurrent density, $\lambda$ the wavelength, and *J*_*Light*_ is the intensity of incident light. The flat band potential was calculated using the Mott–Schottky equation (15 Hz, without light):

4 $$\frac{1}{{C}^{2}}=\frac{2}{{N}_{D}e{\varepsilon \varepsilon }_{0}}\cdot \left[\left({V}_{S}-{V}_{{fb}}\right)-\frac{{k}_{B}T}{e}\right]$$

where *C* is the space-charge capacitance, *V*_*S*_ is the applied potential, *V*_*fb*_ is the flat band potential, *N*_*D*_ is the charge carrier density, *ε* is the relative permittivity of the semiconductor, *ε*_*0*_ is the permittivity of the vacuum, *e* is the elementary charge, and *k*_*B*_ is the Boltzmann constant.

## Supplementary information

Supplementary Information

## Data Availability

Data generated or analyzed during this study are included in this published article and its supplementary information files or are available from the corresponding author on reasonable request.
