# Supplementary material for: Laser-driven growth of structurally defined transition metal oxide nanocrystals on carbon nitride photoelectrodes in milliseconds
Source: Nat Commun. 2021 May 28;12:3224. doi: 10.1038/s41467-021-23367-7 (PMC8163840; doi:10.1038/s41467-021-23367-7)
Supplement: Supplementary file 1 — Supplementary Information [file 41467_2021_23367_MOESM1_ESM.pdf]

# Supplementary Information

**Laser-driven growth of structurally defined transition metal oxide nanocrystals on carbon nitride photoelectrodes in milliseconds**

*Junfang Zhang, Yajun Zou, Stephan Eickelmann, Christian Njel, Tobias Heil, Sebastian Ronneberger, Volker Strauss, Peter H. Seeberger, Aleksandr Savateev, and Felix F. Loeffler**

J. Zhang, Y. Zou, Dr. S. Eickelmann, Dr. T. Heil, S. Ronneberger, Dr. Volker Strauss, Prof. Dr. P. H. Seeberger, Dr. A. Savateev, Dr. F. F. Loeffler
Max Planck Institute of Colloids and Interfaces, Am Muehlenberg 1, 14476 Potsdam, Germany
E-mail: Felix.Loeffler@mpikg.mpg.de

J. Zhang, Prof. Dr. P. H. Seeberger
Department of Chemistry and Biochemistry, Freie Universität Berlin, Arnimallee 22, 14195 Berlin, Germany

Dr. C. Njel
Institute for Applied Materials (IAM) and Karlsruhe Nano Micro Facility (KNMF), Karlsruhe Institute of Technology (KIT), Hermann-von-Helmholtz-Platz 1, 76344 Eggenstein-Leopoldshafen, Germany

| No. | Name | Page |
| --- | --- | --- |
| **1** | **Additional Materials and Methods** | S5 – 7 |
| **Supplementary Figures** | | |
| 2 | **Supplementary Fig. 1** Characterization of carbon nitride films. | S8 |
| 3 | **Supplementary Fig. 2** Preparation of donor slides and acceptor slides. | S9 |
| 4 | **Supplementary Fig. 3** 488 nm LTRAS machine setup showing the different components. | S10 |
| 5 | **Supplementary Fig. 4** Corresponding laser parameters in the 488 nm LTRAS setup for the gradients. | S11 |
| 6 | **Supplementary Fig. 5** Characterization of thin CN layer after transfer. | S13 |
| 7 | **Supplementary Fig. 6** Survey, C 1s, and N 1s XPS spectra of CN and CuO/CN composite films. | S14 |
| 8 | **Supplementary Fig. 7** Evidence for rapid (millisecond) synthesis process with LTRAS. | S15 |
| 9 | **Supplementary Fig. 8** Thickness profile and 3D view of the spots with 0.132 mW/$\mu m^2$ laser power density and 40, 30, and 20 ms irradiation. | S18 |
| 10 | **Supplementary Fig. 9** Proposed growth mechanism for laser transfer. | S19 |
| 11 | **Supplementary Fig. 10** High-resolution TEM images of CuO and proposed mechanism for the laser-driven transfer synthesis. | S20 |
| 12 | **Supplementary Fig. 11** XRD spectrum of CN, NiO/CN, and CoO/CN composite films. | S21 |
| 13 | **Supplementary Fig. 12** SEM image and EDX mapping of the transferred NiO/CN composite film. | S22 |
| 14 | **Supplementary Fig. 13** SEM images and EDX mapping of a spot array on a glass substrate generated with an S-LEC and copper nitrate donor slide. | S23 |
| 15 | **Supplementary Fig. 14** SEM images and EDX mapping of a spot array on an FTO substrate generated with an S-LEC and copper nitrate donor slide. | S24 |
| 16 | **Supplementary Fig. 15** SEM images of single LTRAS spots on carbon-coated glass substrate generated using a S-LEC polymer donor slide. | S25 |
| 17 | **Supplementary Fig. 16** SEM image and EDX mapping of a spot array on a glass substrate, generated with a PVP and copper nitrate donor slide. | S26 |
| 18 | **Supplementary Fig. 17** SEM image and EDX mapping of a spot array on an FTO substrate generated with a PVP and copper nitrate donor slide. | S27 |

| | | |
|---|---|---|
| 19 | **Supplementary Fig. 18** Transient photocurrent response of $CuO_{F1}$/CN electrodes with and without hole scavenger (TEOA) in 0.1 M NaOH solution at 1.23 V *vs.* RHE (reversible hydrogen electrode). | S27 |
| 20 | **Supplementary Fig. 19** Oxygen concentration in the electrolyte, photocurrent density of the composite $CuO_{F1}$/CN electrode, and integration of current with respect to time in the linear region. | S28 |
| 21 | **Supplementary Fig. 20** Increase of photocurrent density in comparison with the steady-state fluorescence intensity. | S28 |
| 22 | **Supplementary Fig. 21** Photograph of synthesis process: CN substrate in $Cu(NO_3)_2$ solution, laser irradiation, CN with spots array. SEM image and EDX mapping of spot generated by laser irradiation. | S29 |
| 23 | **Supplementary Fig. 22** Schematic representation of the photodeposition process on CN substrate. SEM image of CN substrate before and after UV irradiation. EDX spectrum of CN substrate before and after UV irradiation. | S30 |
| 24 | **Supplementary Fig. 23** EDX mapping of rod-like structure on the surface of carbon nitride after 4 h of irradiation with UV. | S31 |
| 25 | **Supplementary Fig. 24** Photoelectrochemical properties of copper-doped carbon nitride obtained by photodeposition. Linear sweep voltammetry curves and chronoamperometry at 1.23 V *vs.* RHE (reversible hydrogen electrode) with or without white light. | S31 |
| 26 | **Supplementary Fig. 25** Schematic representation of copper-doped CN synthesis process by co-polymerization. EDX spectrum of $Cu_1$-CN and $Cu_{10}$-CN. | S32 |
| 27 | **Supplementary Fig. 26** Photograph and TEM image of $Cu_{0.1}$-CN. Photograph of the electrodes prepared using doctor blade technique. Linear sweep voltammetry curves of the electrodes. Chronoamperometry of electrodes at 1.23 V *vs.* RHE. | S33 |
| 28 | **Supplementary Fig. 27** Photocurrent density of pristine CN and composite $CuO_{F1}$/CN electrodes under irradiation with different light intensity. The corresponding linear fittings between current densities and incident light intensities are shown on the right side. | S34 – 35 |
| 29 | **Supplementary Fig. 28** Chronopotentiometry of $CuO_{F1}$/CN electrodes under 50 μA constant current with blue light irradiation. | S36 |
| 30 | **Supplementary Fig. 29** Photocurrent response of CuO/CN and CN electrodes to glucose injection under white light or blue light in 0.1 M NaOH solution. | S37 |
| 31 | **Supplementary Fig. 30** Tauc plots of CN and CuO. Mott-Schottky plots of CN, CuO, and $CuO_{F1}$/CN electrodes at 15 Hz. | S38 |
| 32 | **Supplementary Fig. 31** Nyquist plots for CN, $CuO_{R2}$/CN (single-layer CuO nanorods) and $CuO_{R3}$/CN (multi-layer CuO nanorods). | S39 |

| | | |
|---|---|---|
| 33 | **Supplementary Fig. 32** UV-visible absorption spectra of CN and CuO electrodes. | S39 |
| 34 | **Supplementary Fig. 33** 405 nm LTRAS machine setup showing the different components. | S40 |
| 35 | **Supplementary Fig. 34** Vertical scanning interferometry (VSI) measurement of donor slide and thickness profile. | S41 |
| 36 | **Supplementary Fig. 35** Measured intensity vs. wavelength spectra of the white (300 $mW/cm^2$) and blue (100 $mW/cm^2$) LED lamps. | S41 |
| **Supplementary Tables** | | |
| 37 | **Supplementary Table 1** Summary of CuO nanostructures obtained by different methods. | S42 |
| 38 | **Supplementary Table 2** PEC performances of recent publications involving CN photoanodes. | S43 |
| 39 | **Supplementary Table 3** List of copper-based non-enzymatic glucose sensors. | S44 |
| 40 | **Supplementary Table 4** Effective absorbed laser energy fluences for the two laser setups at different laser powers | S45 |

## Additional Materials and Methods

### Preparation of PVP-based donor slides

80 mg of PVP (polyvinylpyrrolidone, av. *M*w ≈1 300 000, Sigma) and 80 mg of $Cu(NO_3)_2 \cdot xH_2O$ (99 %, Acros) were dissolved in 500 μl of dd$H_2O$. We spin-coated the solution on polyimide film (Kapton HN 100 type, Dupont, USA) covered glass slides at 80 rps.

### Cu-based CN electrodes from photodeposition

270 mg of $Cu(NO_3)_2 \cdot xH_2O$ (99 %, Acros) were dissolved in 3 ml dd$H_2O$ in a petri dish (⌀ 25 mm), and a piece of FTO glass (7 Ω/sq, Merck) with a CN layer was immersed in this precursor solution (Supplementary Fig. 21). Then, the petri dish was placed under a laser system (1.9 W, 450 nm) and irradiated to create a 10 x 10 pattern (full power, 100 – 450 ms). SEM (Supplementary Fig. 21b) and EDX mapping show that no CuO was formed in this process even though the effective laser power was more than 70 times higher than that in the LTRAS method (1.9 W @ 450 nm *vs.* 25 mW @ 405 nm). These results indicate that the well-established photodeposition method needs much more energy and/or time than the LTRAS method. Therefore, we switched to a UV light approach together with a longer irradiation time (Supplementary Fig. 22). A piece of CN substrate was immersed in 0.1 M $Cu(NO_3)_2 \cdot xH_2O$ solution under UV light for several hours (1 h, 2 h, or 4 h). The photoelectrochemical performance was measured in a three-electrode cell. Pt electrode, Ag/AgCl in saturated KCl, and 0.1 M NaOH solution were used as the counter electrode, reference electrode, and electrolyte respectively.

### Cu-based CN electrodes from co-polymerization

0.1wt.%, 1wt.%, or 10wt.% $Cu(NO_3)_2 \cdot xH_2O$ (99 %, Acros) and 5 g melamine (99 %, Alfa Aesar) were added in 50 ml dd$H_2O$ and stirred at 80 °C for 2 h. Afterwards, we removed the

water by centrifugation (8000 rpm, 2 min). The obtained material was dried in an oven at 80 °C and labeled as “Cu-M”. We put the Cu-M into a crucible and calcined in a furnace (570 °C, 3 h). Then, the powder was washed three times with $ddH_2O$ to remove possible remaining precursor and intermediates with lower polymerization degree. According to the amount of Cu precursor, the final powder is labeled as $Cu_{0.1}$-CN, $Cu_1$-CN, or $Cu_{10}$-CN. In the next step, we deposited these pre-synthesized powders on the electrodes. Briefly, 2 mg of $Cu_{0.1}$-CN, $Cu_1$-CN, or $Cu_{10}$-CN and 20 µl Nafion solution (5 wt. %, Aldrich) were mixed with 200 µl of $ddH_2O$. After ultrasound treatment for 1 h, we deposited the suspension on the FTO substrate by doctor blading. All electrodes were prepared with a 1 cm x 1 cm area of the light-responsive $Cu_x$-CN material (Supplementary Fig. 25). The photoelectrochemical performance was measured in a three-electrode cell. Pt electrode, Ag/AgCl in saturated KCl, and 0.1 M NaOH solution were used as the counter electrode, reference electrode, and electrolyte respectively.

**IPCE measurements**

A series of fiber-coupled LEDs (SMA, ThorLabs) with wavelengths of 415 nm, 455 nm, 530 nm, 625 nm, and 850 nm were connected to the LED Driver (DC2200, ThorLabs) and used as light sources for the IPCE measurements. For each wavelength, we studied the dependence of current density versus intensity of the incident light (5 data points) (Supplementary Fig. 27). The current density depends linearly on the intensity of the incident light. The slope of linear fitting corresponds to the ratio between $J_{Ph}$ and $J_{Light}$.

**Glucose detection by amperometry**

The response of the pristine CN and the composite $CuO_{F1}$/CN electrodes to glucose was evaluated using amperometry by successively adding small amounts of concentrated glucose solution. In detail, the phototelectrochemicial performance was assessed using a three-electrode cell. Pt electrode, Ag/AgCl in saturated KCl and 0.1 M NaOH solution were used as counter

electrode, reference electrode, and electrolyte respectively. We added 100 µl of glucose solution into 20 ml of 0.1 M NaOH electrolyte for each 30 s intervals. The concentration of glucose solution used in this experiment were 10 mM, 10 mM, 20 mM, 20 mM, and 40 mM, respectively (Supplementary Fig. 29). In the electrolyte, we obtained glucose concentrations in the range from 0.05 to 0.5 mM. The measurements were performed at 0.1 V *vs.* Ag/AgCl under white light (300 mW/cm$^2$) or blue light (100 mW/cm$^2$).

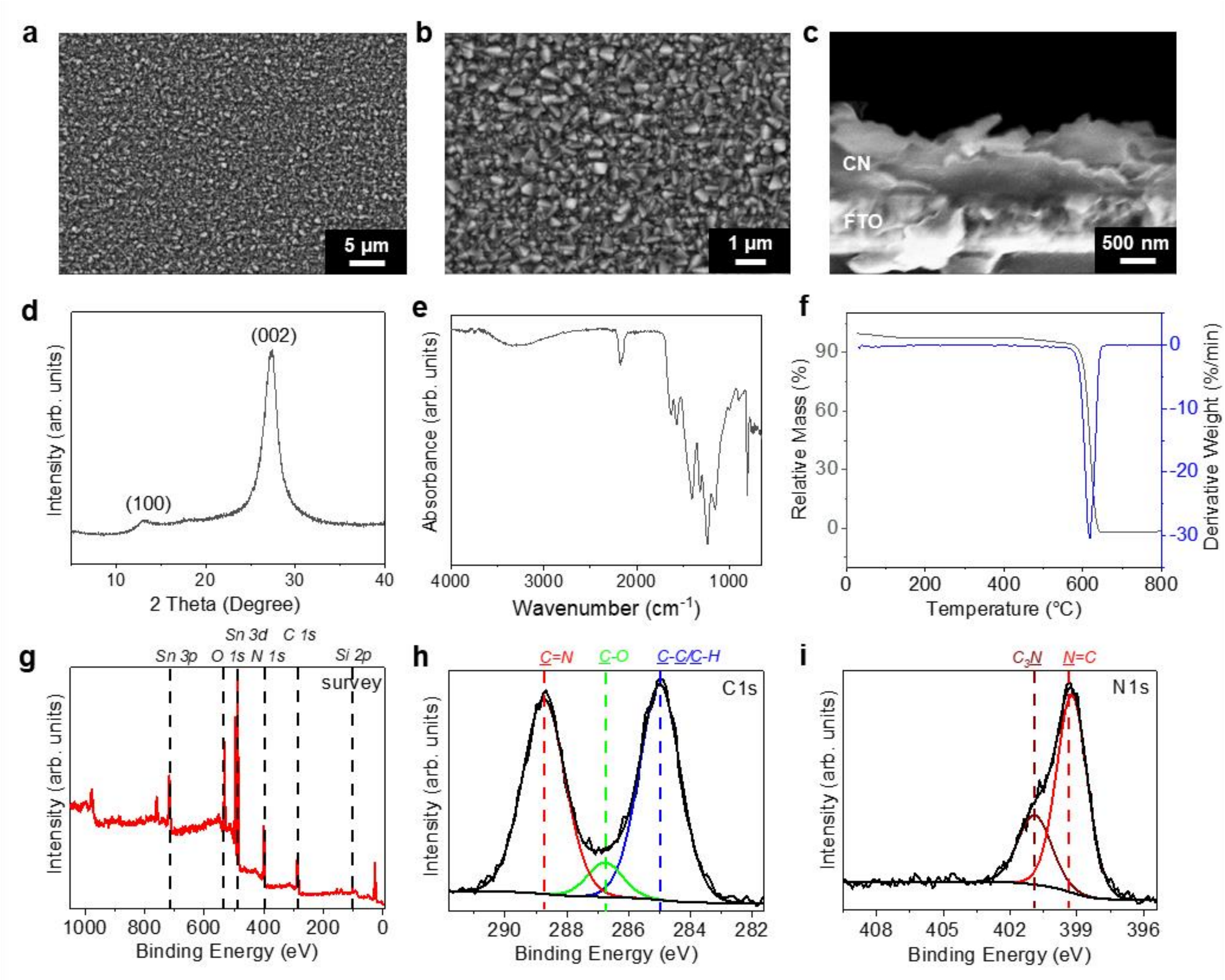


**Supplementary Fig. 1** Characterization of carbon nitride films. (a, b) SEM image and (c) cross-section image of carbon nitride (CN) layer on the FTO electrode. (d) XRD pattern, (e) FTIR spectrum, (f) TGA curve, and (g, h, i) XPS spectrum of CN layer.

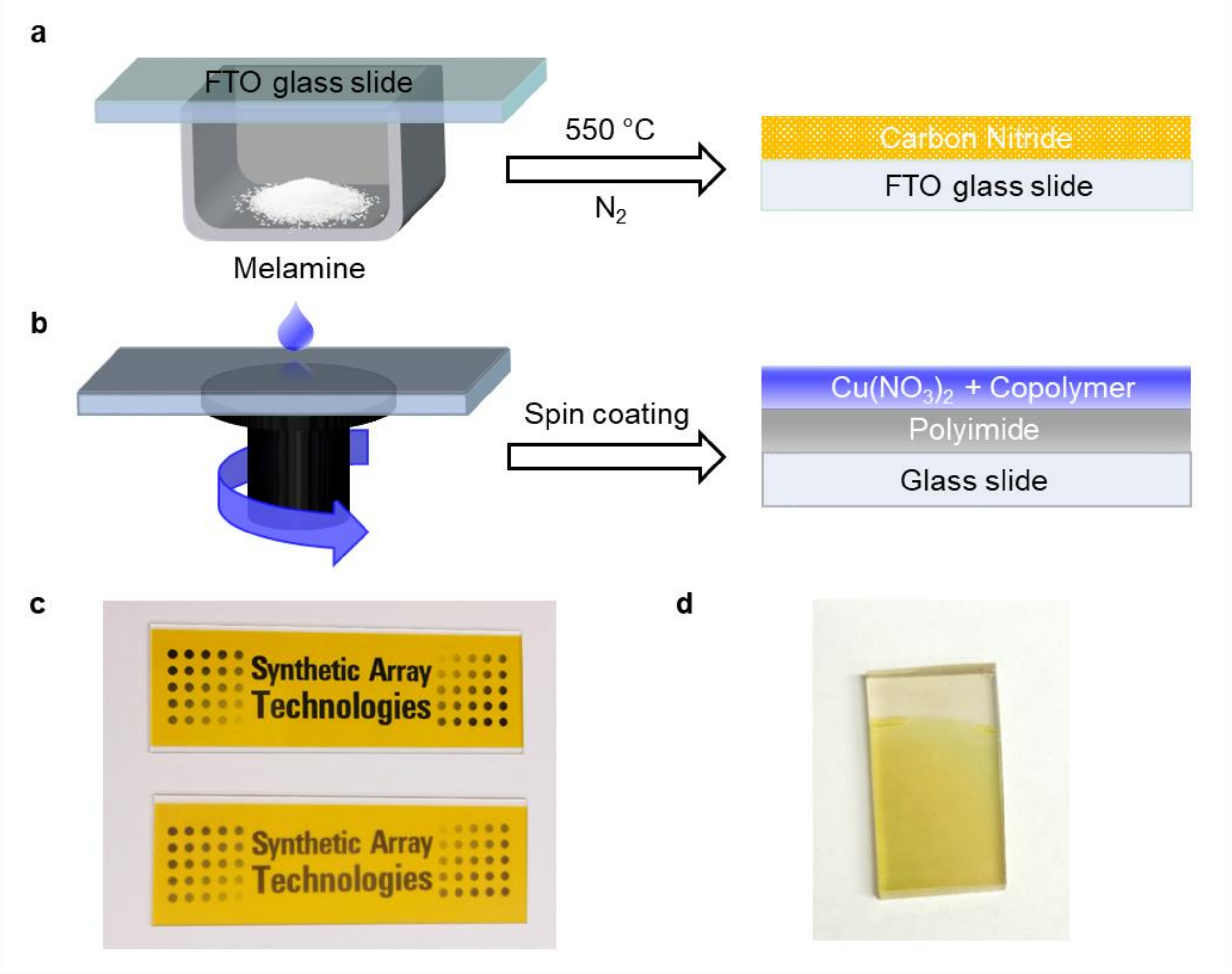


**Supplementary Fig. 2** Preparation of donor slides and acceptor slides. (a) An acceptor slide is prepared by vapor deposition polymerization of CN onto an FTO glass slide. (b) Preparation of a donor slide by spin coating a mixture of dissolved Cu nitrate ($Cu(NO_3)_2$) with a copolymer onto a polyimide-coated glass slide. (c) Kapton slide before (top) and after (bottom) spin coating. (d) Photograph of acceptor slide with the CN film (yellow).

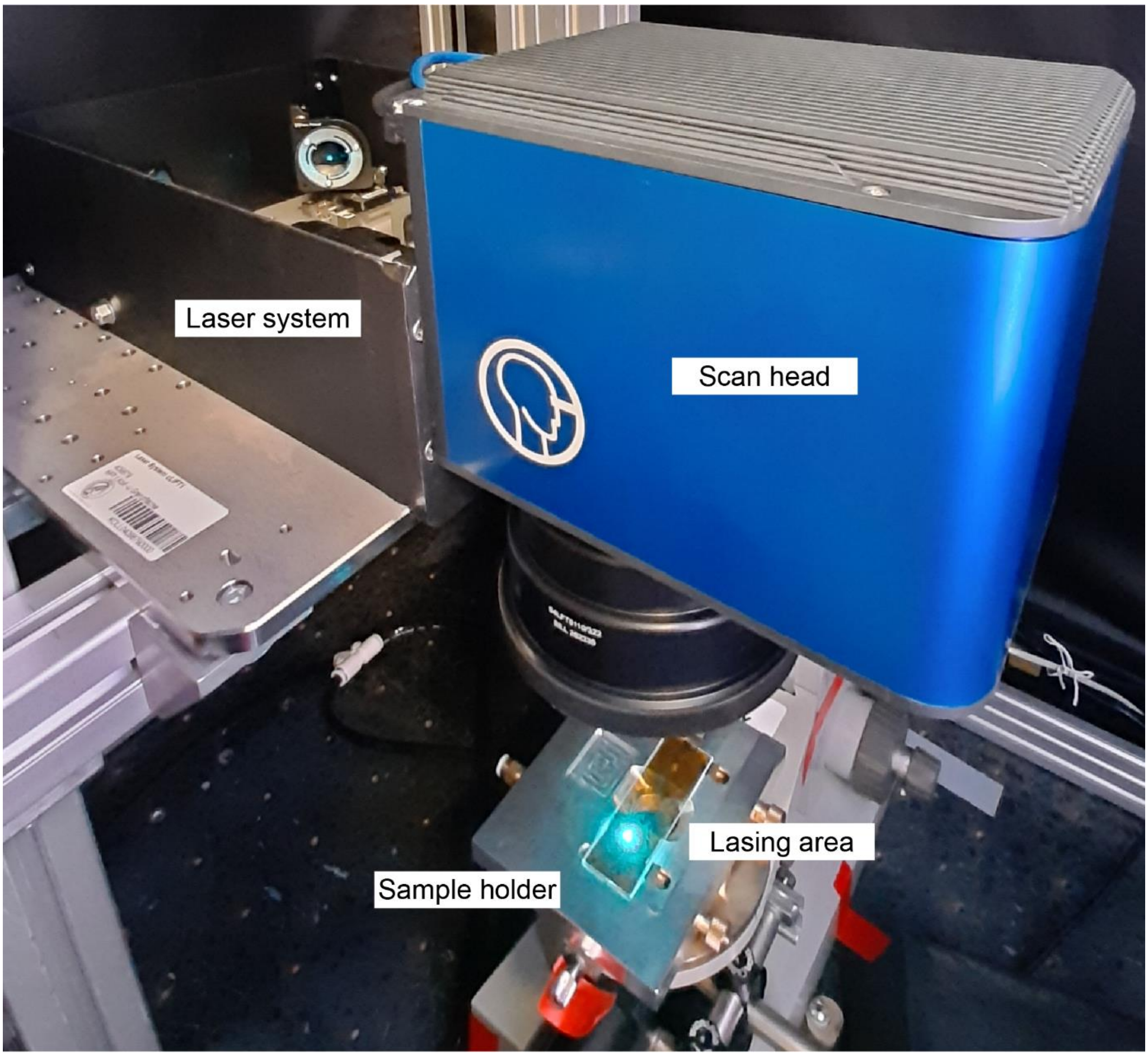


**Supplementary Fig. 3** 488 nm LTRAS machine setup showing the different components.

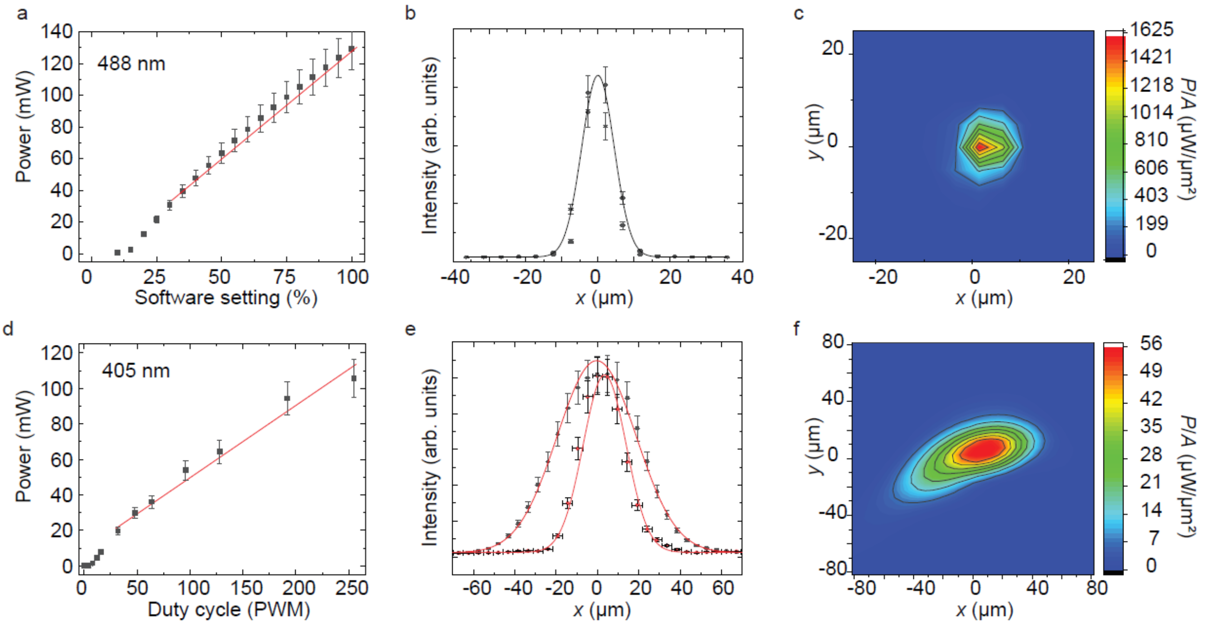


**Supplementary Fig. 4** Laser output power (a, d) with standard deviation (of 30 s average) and laser profiles (b, c, e, f) with measurement uncertainty. Calibration curves of power vs. software settings in percentage (a) for the 488 nm system and (d) duty cycle for the 405 nm system (power meter PM200, Thorlabs). Laser profiles of the (b, c) 488 nm system and the (e, f) 405 nm system were determined with a CMOS sensor (xiQ USB3 Vision MQ013MG-ON, Ximea GmbH) with a pixel resolution of 4.8 µm, calibrated with the power meter. The lateral energy distribution inside the focal points (b, e) are shown. The 488 nm focus (b) can be easily fitted to a Gaussian profile with a spot size of $1/e^2 = 18$ µm. The 405 nm laser diode has an elliptical profile (e, f), thus two diameters can be extracted (42 & 77 µm). Since we move the laser along the x-axis during the process, we assume a Gaussian beam with a diameter of $1/e^2 = 42$ µm.

We use continuous wave (cw) laser setups in the line scanning mode with speeds between 16.7 – 60 µm/ms. Usually, the differentiation between pulsed and cw mode is around 10^-6 s, where ablation effects start to dominate, which is why we consider our approach cw, although we not only used line scanning, but also ms pulses for the experiment in Figure 3. To validate our transfer with different energies, we used the spot-mode, where we irradiated single spots for longer times (20 – 40 ms and 61.8 – 90 mW), as shown in Figure 3.

Calculations for cw laser with continuous movement speed:

Effective irradiation time: $\boldsymbol{\Delta t_{EIR}} = \frac{\boldsymbol{d_{spot}}}{\boldsymbol{v_{laser}}}$

Peak power density for Gaussian beam [J/s*cm$^2$]:

$$\frac{\mathbf{2 \cdot Power} \left[\frac{J}{s}\right]}{\frac{\pi}{4} \cdot d_{spot}^2 \, [cm^2]}$$

Effective (absorbed) fluence [J/cm$^2$]:

$$\boldsymbol{Power\ density}\ \left[\frac{\boldsymbol{J}}{\boldsymbol{s}\cdot \boldsymbol{cm^2}}\right]\cdot \boldsymbol{Absorption_{wavelength}}[\%]\cdot \boldsymbol{\Delta t_{EIR}}[\boldsymbol{s}]$$

488 nm:

The 488 nm laser spot was used with 35 – 60 µm/ms scanning speed and has a Gaussian spot profile with the 1/e² size of $d_{spot,488nm}$ = 18 µm. Thus, the effective irradiation time is between 0.30 ms and 0.51 ms. The absorption of our polyimide coated donor slide according to Figure 3 is 22 % at 488 nm. The power density at P = 90 mW is 7.1·10^4 J/(s·cm^2) and at 76.4 mW it is 6.0·10^4 J/(s·cm^2). The typical scanning line density was 12.5 µm. With a laser focus diameter of 18 µm (1/e²) and a FWHM of ~12 µm, we can assume that each area is hit by peak laser irradiation only once.

405 nm:

The 405 nm system has a writing speed of 16.7 µm/ms and an elliptical profile with two different laser spot sizes (1/e²) in x and y direction of $d_{x\text{-}spot,405nm}$ = 42 µm and $d_{y\text{-}spot,405nm}$ = 77 µm. This results in an effective irradiation of 4.6 ms, since we scanned in the y direction.
Yet, since the writing direction matches the x-axis of the laser profile, we roughly assume the smaller diameter as the spot size for calculating the energy density. A line density (20 per mm) of 50 µm spacing was used. Since the laser spot diameter in x direction is 42 µm, each area is hit only once by the laser. With the approximation of a Gaussian laser profile, the maximum power density is around 6·10^3 J/(s·cm^2) at an optical power output of P = 42 mW and 3.6·10^3 J/(s·cm^2) at 25 mW. The absorption of our polyimide coated donor slide according to Figure 3 is maximum (~100 %) at 405 nm.

These assumptions result in effective fluences, shown in Supplementary Table 4.

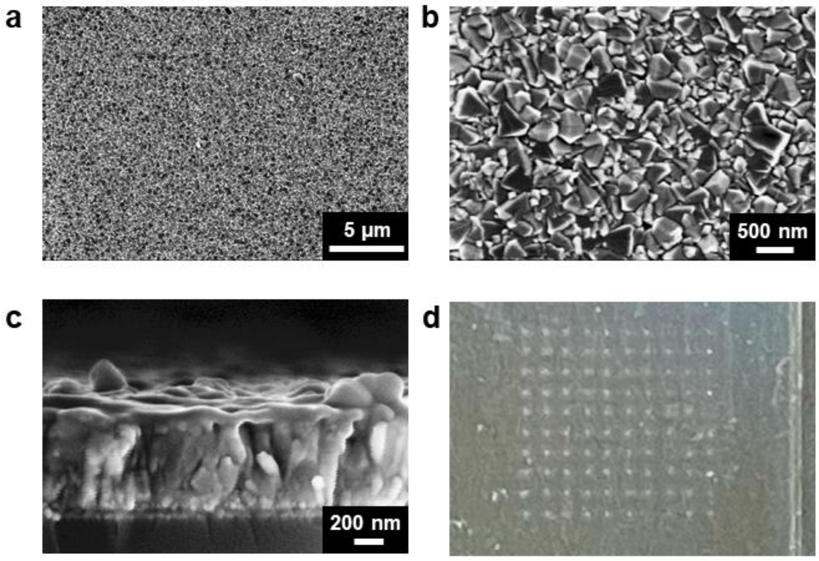


**Supplementary Fig. 5** (a, b) SEM images and (c) cross-section image of a 50 nm thin carbon nitride (CN) layer. (d) Photograph of CN layer after LTRAS of a spot pattern.

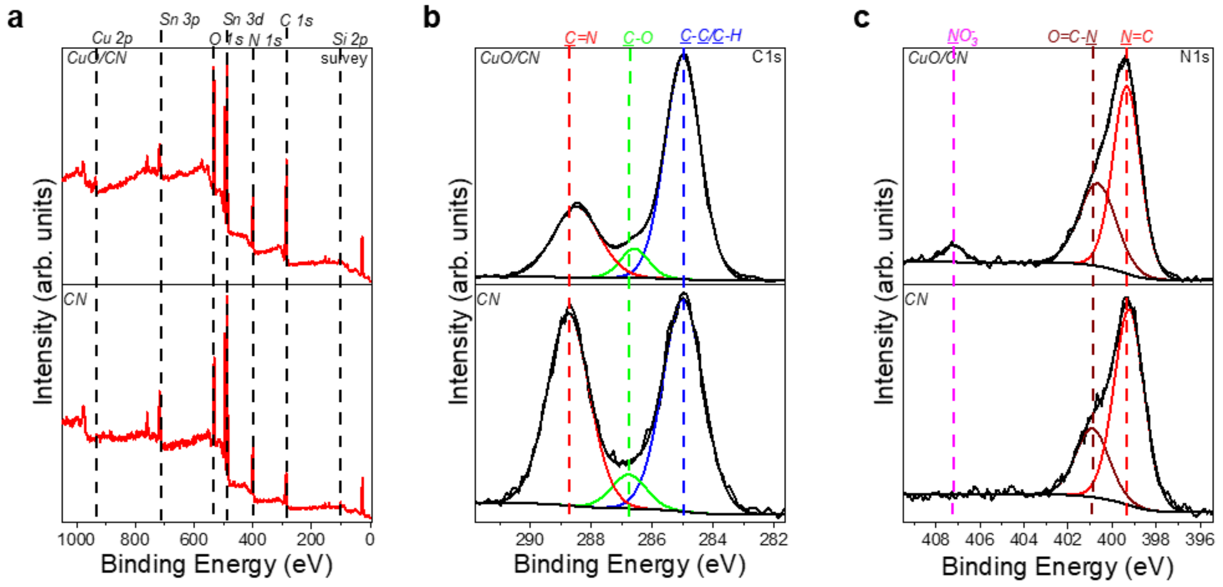


**Supplementary Fig. 6** (a) Survey, (b) C 1*s*, and (c) N 1*s* XPS spectra of (top) CuO/CN composite film and (bottom) pristine CN film.

Survey XPS spectra reveal the presence of six elements such as carbon (C 1*s*), oxygen (O 1*s*), nitrogen (N 1*s*), copper (Cu 2*p*), tin (Sn 3*d* or Sn 3*p*), and silicon (Si 2*p*). The presence of Sn 3d on the survey spectra suggests that both layers are rather thin.

The C 1*s* spectrum shows signals coming from all carbon species on the surface. Three main environments are detected: The blue peak at 285 eV corresponds to carbon from hydrocarbon or C-C bonds, which come from contamination (pristine CN film) and S-LEC copolymer (CuO/CN composite film). The peak around 286.5 eV (green peak) can be attributed to C-O bonds. The peak centered at 288.3 eV (red peak) corresponds to carbon from C=N bonds from the carbon nitride film. In the N 1*s* spectra, three peaks are presented: The peak at 407.2 eV corresponds to Nitrogen from $Cu(NO_3)_2$. The peaks centered at 399.3 eV and 400.7 eV correspond to N=C and O=C-N environments respectively.

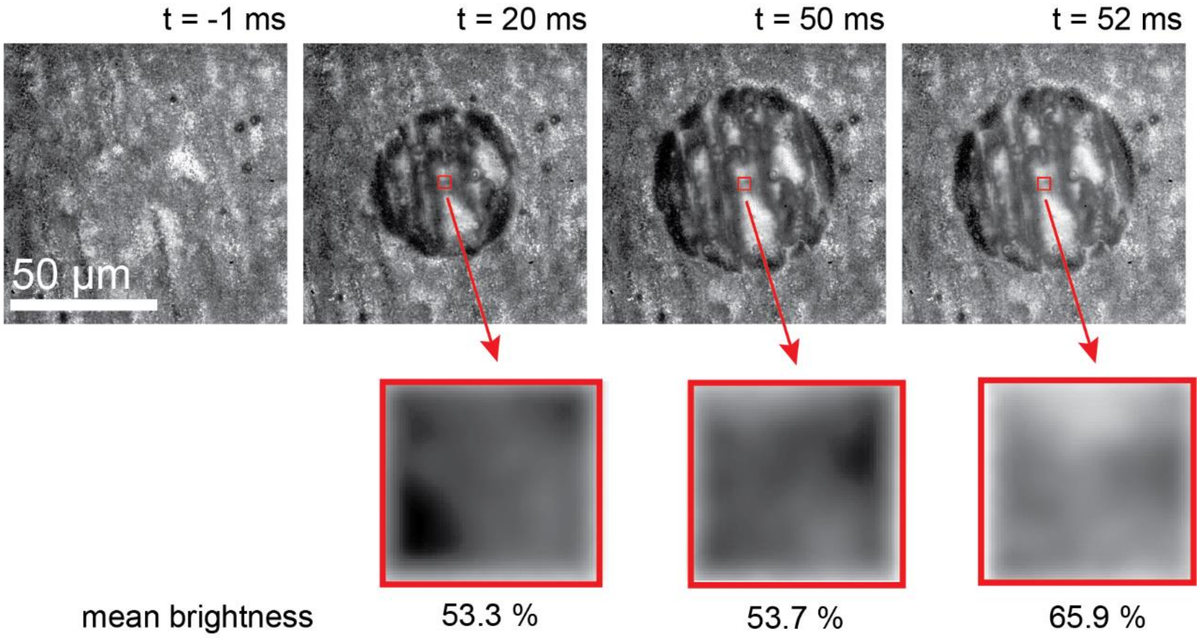


**Supplementary Fig. 7** Melting of a 200 nm thin polymer film, irradiated with 25 mW for 50 ms. Before the laser irradiation (-1 ms), during the laser heating (20 ms), shortly before laser off (50 ms), and after the laser is turned off (52 ms). Analyzing the central brightness value during the process, the value stays constant while the laser is on. After switching the laser off, only 2 ms after the value increases by 10 % and then stays constant. This correlates with the solidification of the polymer.

Our analyses corroborate that the complete process/growth time is in the millisecond regime.

As described before, the local laser interaction is in the range of (sub-)milliseconds (0.33 – 2.4 ms). The energy is absorbed in the µm thick polyimide (PI) film. The polymer matrix with the precursor is only about 1 µm thick, thus axial (in laser beam direction) heat diffusion can be neglected. The process is thus dominated by thermal gradients induced in the PI film.

In the main Figure 3a,b we have shown a typical thermal gradient induced by a local laser irradiation for a comparably long laser irradiation (20 – 40 ms). The laser heating in axial direction can be assumed as nearly instantaneous. Yet, laterally, it will take some milliseconds to heat up the material.

Once the laser irradiation stops, the polymer quickly cools down again. This happens quite fast due to the heat capacity of the acceptor slide.

To prove this, we followed a spot irradiation with a high-speed camera (Supplementary Figure 7), showing that the polymer matrix, which is irradiated for 50 ms with a laser power of

25 mW (405 nm laser) cools down below the melting temperature of the polymer of 210 °C within less than 3 ms.

The optical appearance of a solid polymer film solidifying during the film preparation from solvent (the morphology is characterized with small dewetting holes and other drying effects) and the same film solidified (frozen) due to a temperature related phase transition can easily be distinguished.

The frozen organic film on the other hand is harder to distinguish from the liquid phase, since it rapidly freezes the molten morphology in place. If it would freeze more slowly, other characteristic morphologies can appear.[1]

The polymer solidification is measured by analyzing the local brightness value inside the molten spot. 3 ms after the laser is turned off, there is an increase by 10 %, which only varied by 1 % during the next hundreds of ms (data only shown for 52 ms time point). Since the laser wavelength is filtered out in the optical microscope, we ruled out that it could be the laser (in fact, the brightness value increases after the laser is off, thus it cannot be the laser irradiation, which would cause a decrease of the grey value upon switching off).

Thus, we conclude that after a 50 ms long laser irradiation, the molten polymer spot requires only about 1 – 3 ms to cool down below its melting temperature of around 210 °C after heating to about 400 – 500 °C in the center.

This means, for a shorter irradiation time, the cooling time should be even shorter. Taking this into account, total process time (limited by the molten polymer) is in the range of about 1 – 5 ms. Once the polymer is “solid” again, the CuO cannot freely move and nucleate anymore.

Concluding, the LTRAS mechanism for the formation of the CuO is based on the phase transition of the polymer matrix. As long as the polymer is “frozen/solid” the CuO cannot form. Once it melts, the CuO can form and nucleate. Since heterogeneous nucleation is always favored, this will happen at the acceptor surface interface. Additionally, the cooler interface will lower the energy barrier and favor nucleation. Considering that the process window is

defined by the polymer in its liquid state, the process needs a temperature above 210 °C. Thus, the process is very fast and in the millisecond regime.

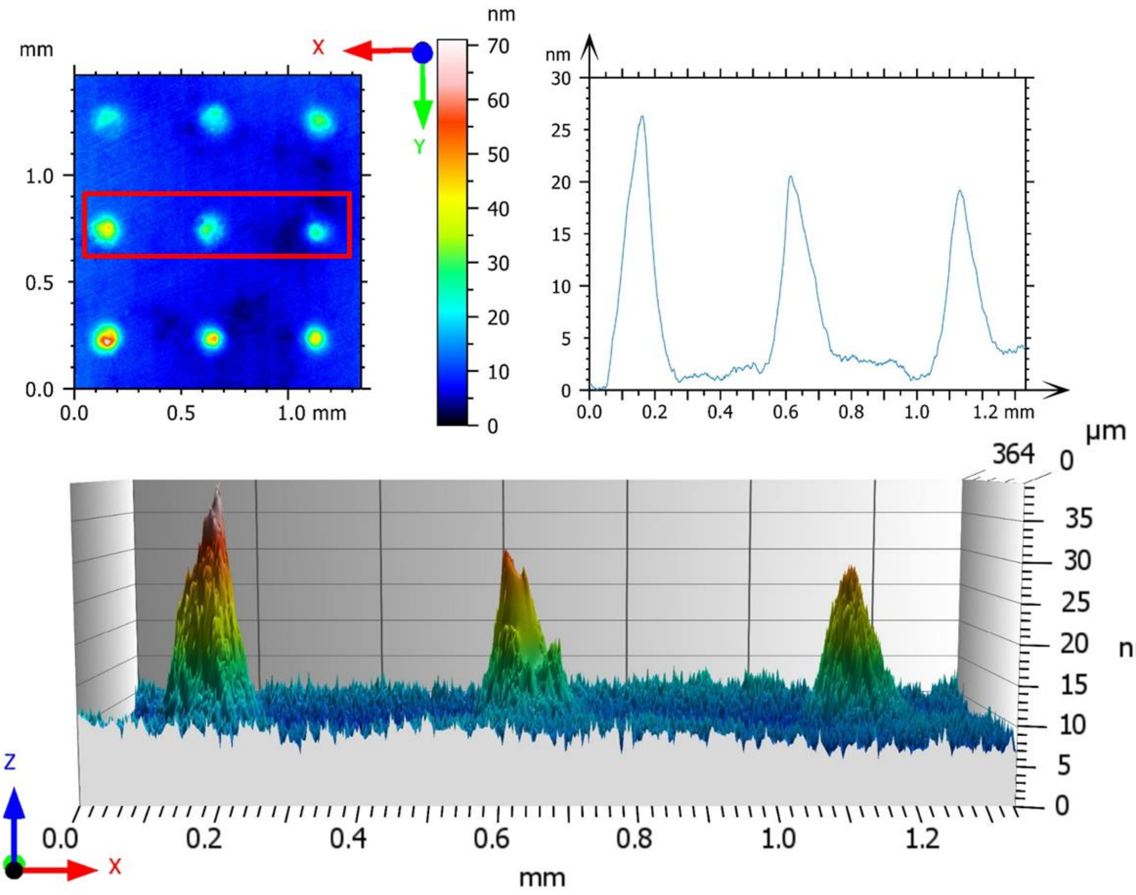


**Supplementary Fig. 8** Thickness profile and 3D view of the spots with 0.132 mW/µm² effective laser power density and 40, 30, and 20 ms irradiation.

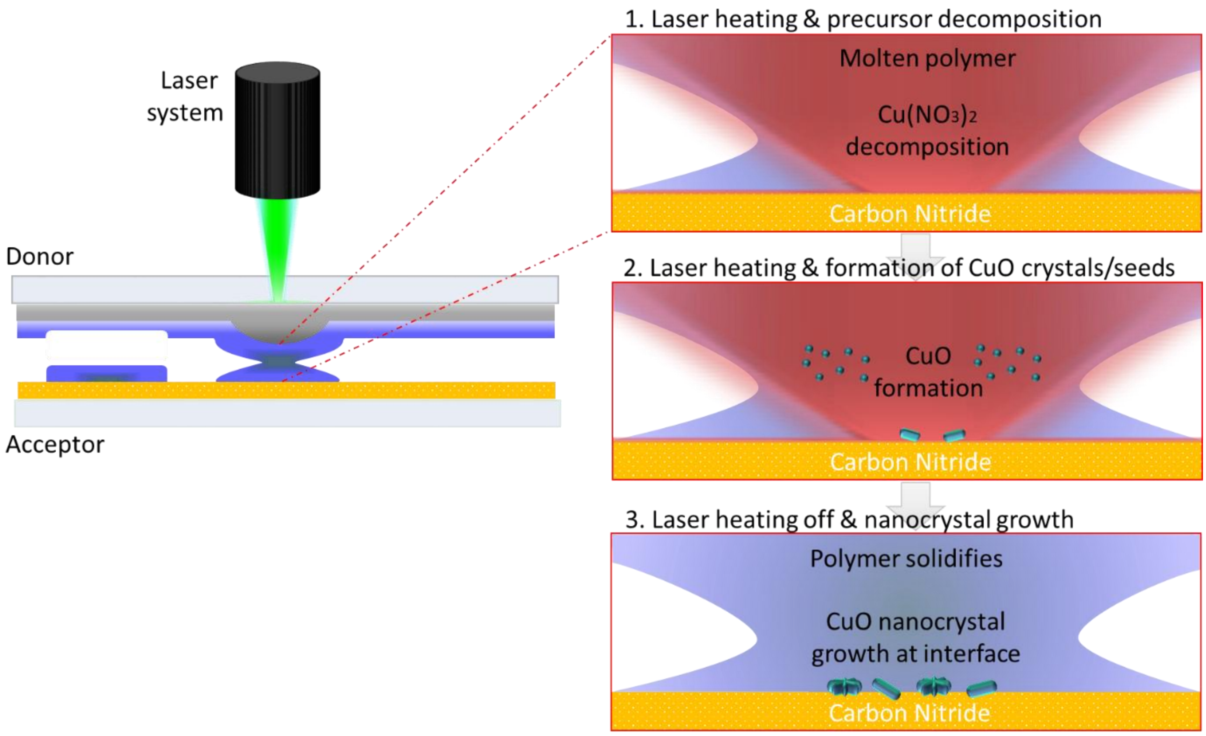


**Supplementary Fig. 9** Proposed growth mechanism, based on three steps: (1) precursor decomposition, (2) growth at the interface, and (3) solubility differences in the molten vs. solid polymer.

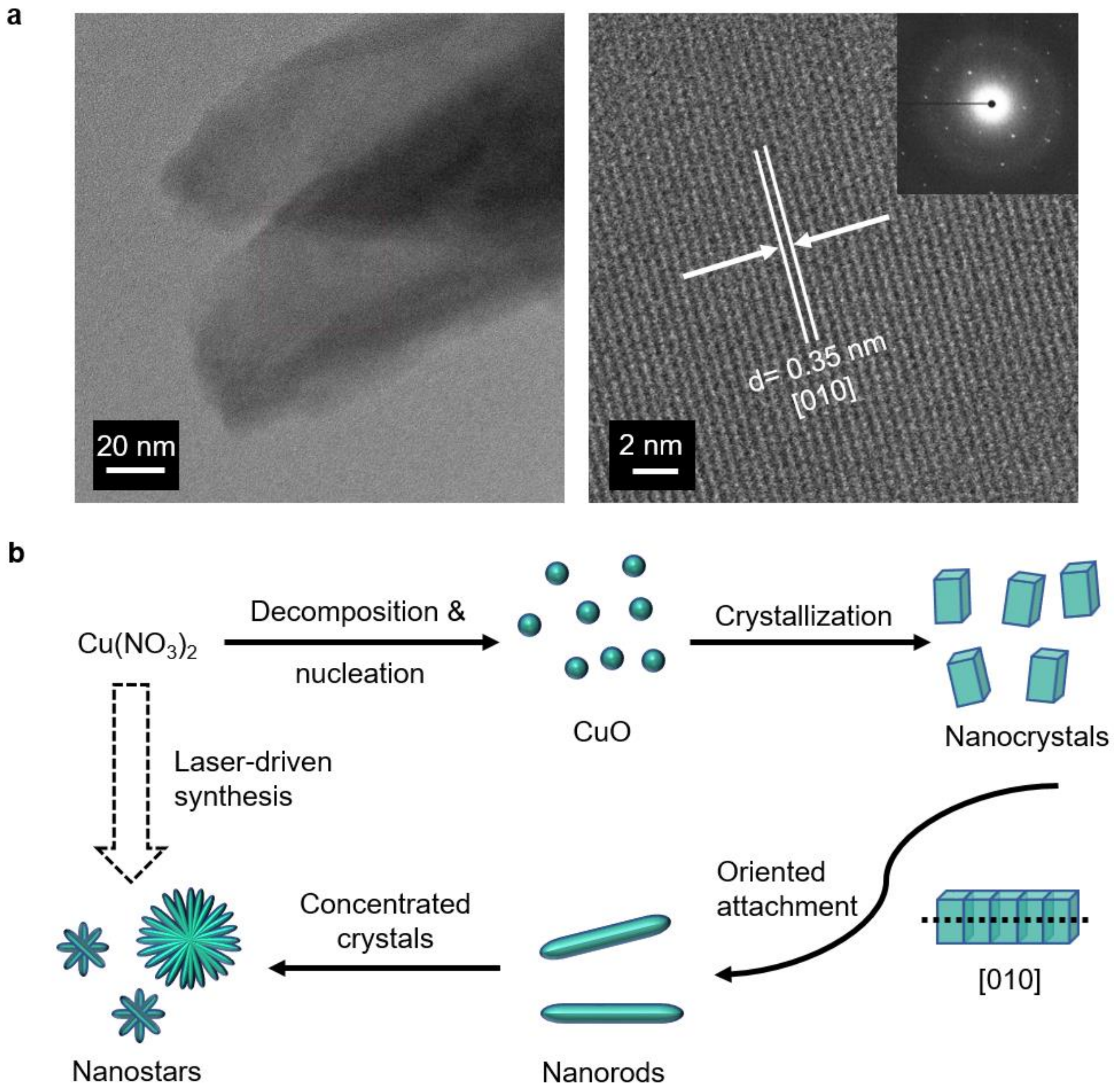


**Supplementary Fig. 10** (a) High-resolution TEM images of CuO. The inset shows the diffraction pattern with selected area electron diffraction (SAED). (b) Proposed mechanism for the laser-driven transfer synthesis.

To reveal the mechanism of laser-driven synthesis, high-resolution transmission electron microscopy (TEM) and selected area electron diffraction (SAED) were performed for the LTRAS-obtained CuO. In these images, a highly ordered crystalline structures of CuO can be clearly observed. The fringe spacing is measured to be approximately 0.35 nm, which corresponds to the [010] lattice fringe of the monoclinic CuO. This result suggests that the preferred growth direction of the nanorods is [010]. Accordingly, we propose the following

mechanism: The synthesis starts from the decomposition of Cu nitrate, which follows the typical process steps of nucleation and growth.[2] First, during the growth step nanocrystals are formed. In the second stage, the obtained nanocrystals arrange into nanostructures.[3] Here, we propose the well-established theory of ‘oriented attachment’.[4] Small crystallites attach to each other *via* their related crystal facets along the same directions, forming larger crystals.[5] Based on the results from TEM and SAED, facet [010] is the preferred growth direction of the CuO nanocrystals. Thus, the final rods can be considered as large crystals composed of pristine crystallites in a highly oriented manner. Assuming higher concentration of CuO nanocrystals in the mixture at slower scanning speed, the probability of faults, i.e. attachment of the nanocrystal to a wrong facet, becomes higher, and therefore star-like nanostructures are formed under such conditions.

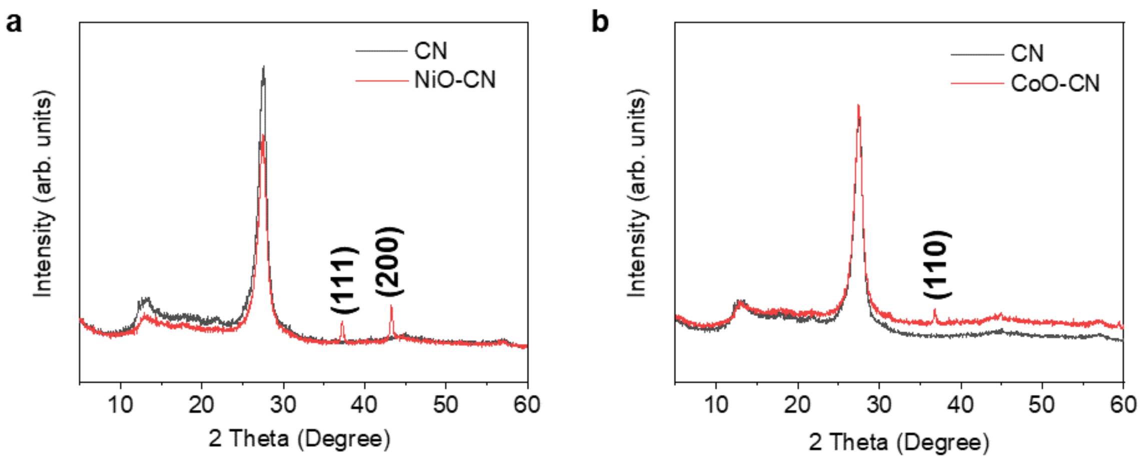


**Supplementary Fig. 11** XRD spectrum of (a) CN and NiO/CN composite film, (b) CN and CoO/CN composite film.

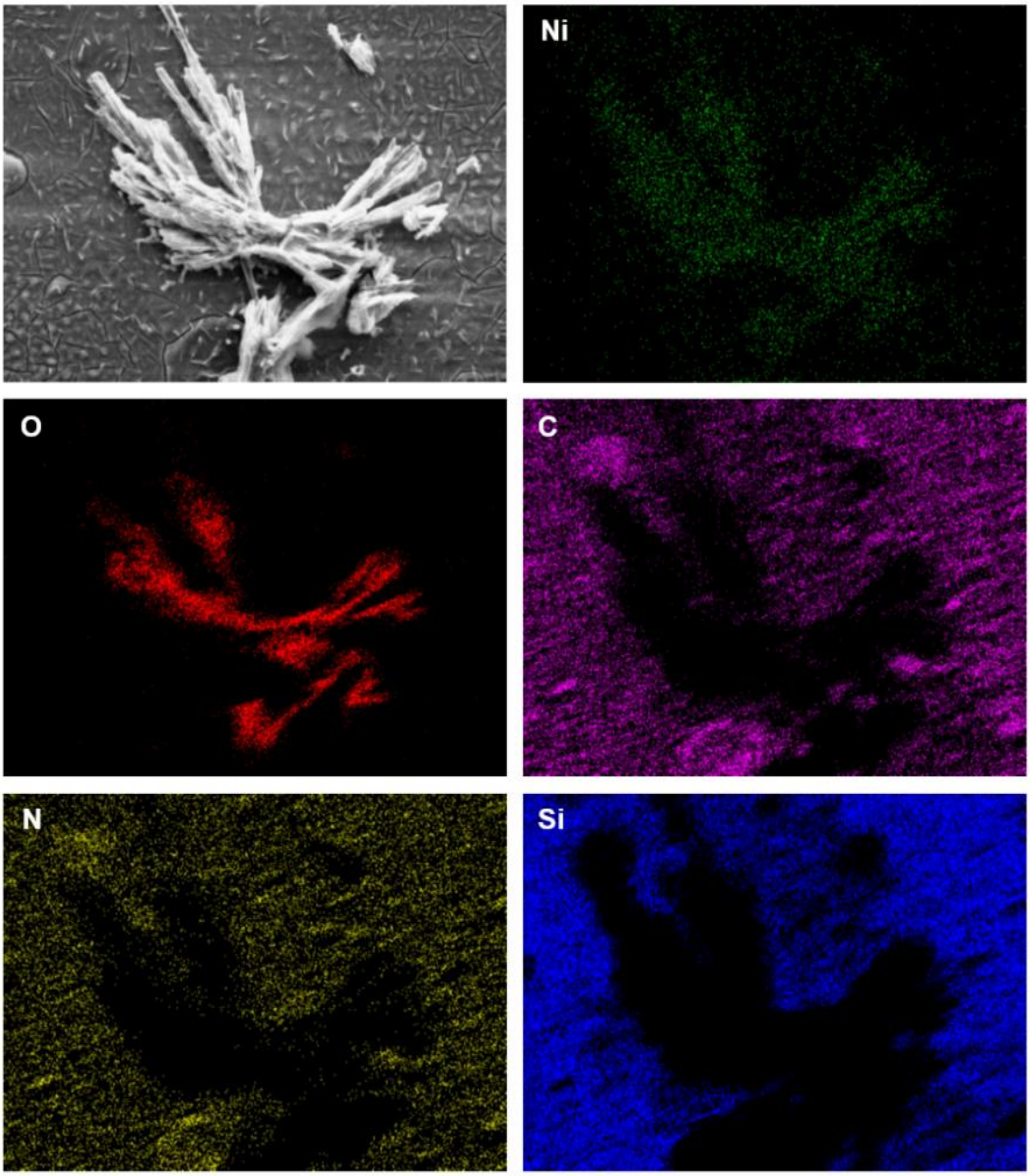


**Supplementary Fig. 12** SEM image (top left) and EDX elemental maps of the transferred NiO/CN composite film.

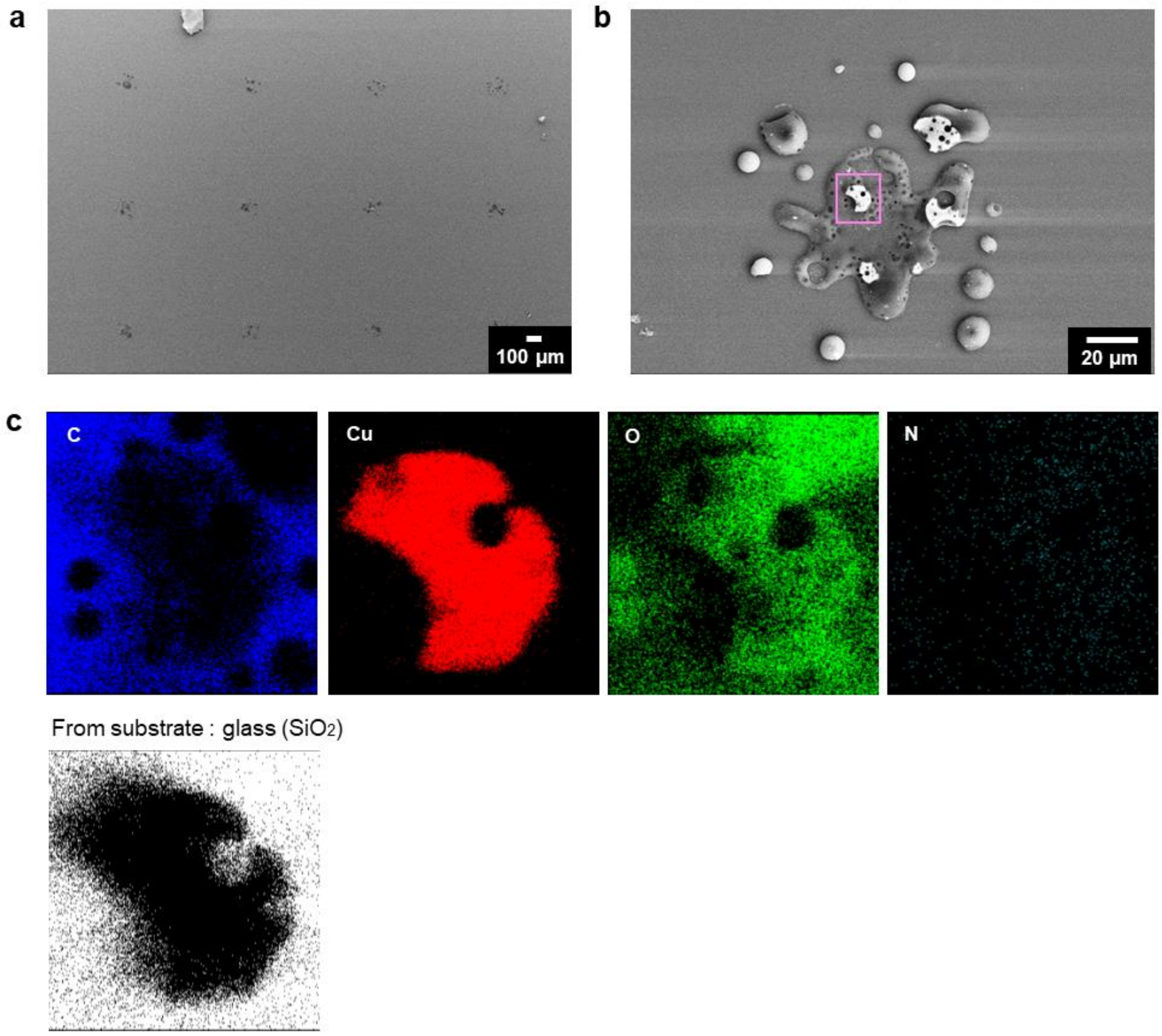


**Supplementary Fig. 13** (a, b) SEM images and (c) EDX mapping of a spot array on a glass substrate generated with an S-LEC and copper nitrate donor slide.

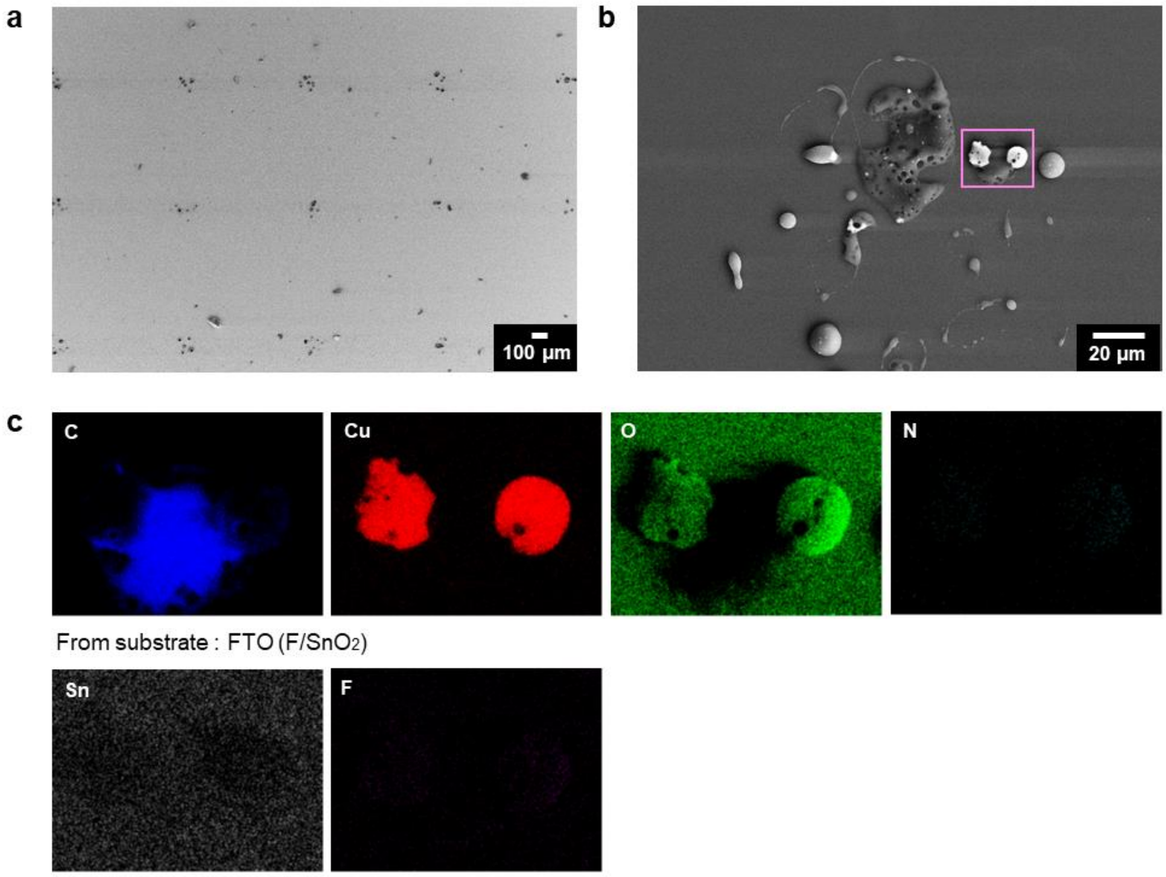


**Supplementary Fig. 14** (a, b) SEM images and (c) EDX mapping of a spot array on an FTO substrate generated with an S-LEC and copper nitrate donor slide.

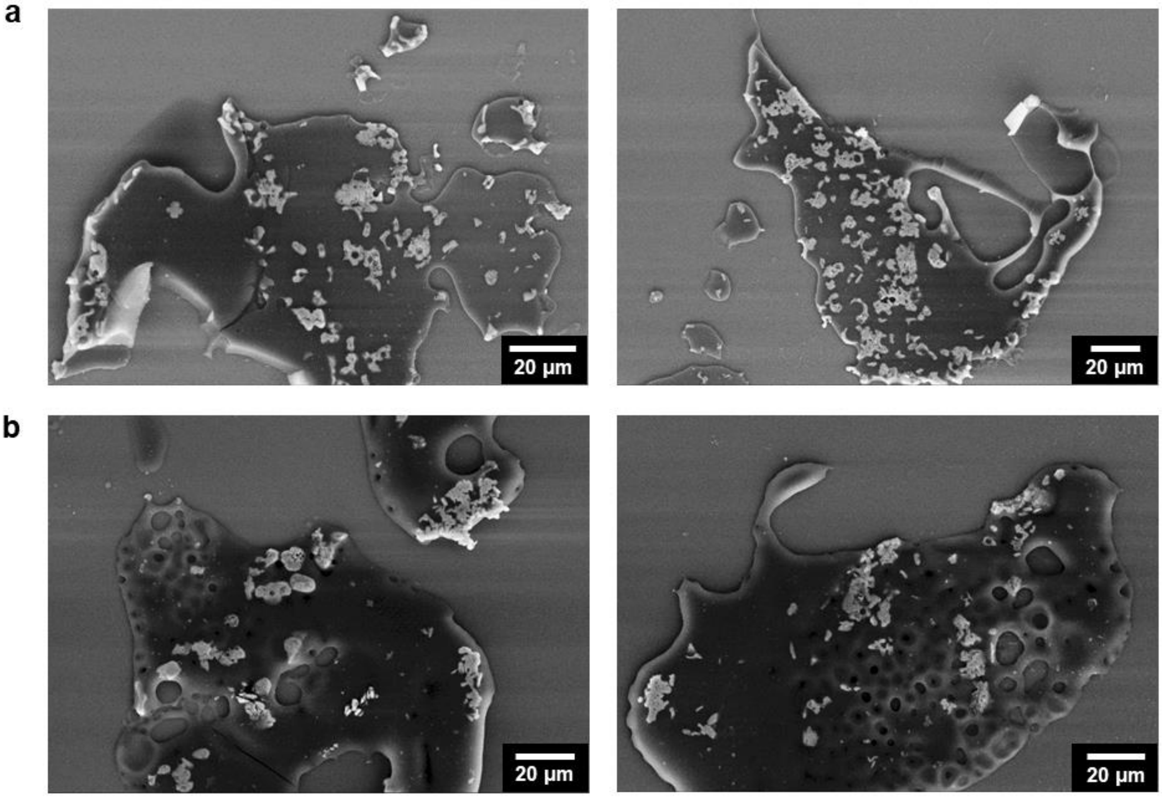


**Supplementary Fig. 15** SEM images of single LTRAS spots on carbon-coated glass substrate generated using a S-LEC polymer donor slide. (a) Low energy (0.132 mW/µm², 300 ms). (b) High energy (0.174 mW/µm², 300 ms).

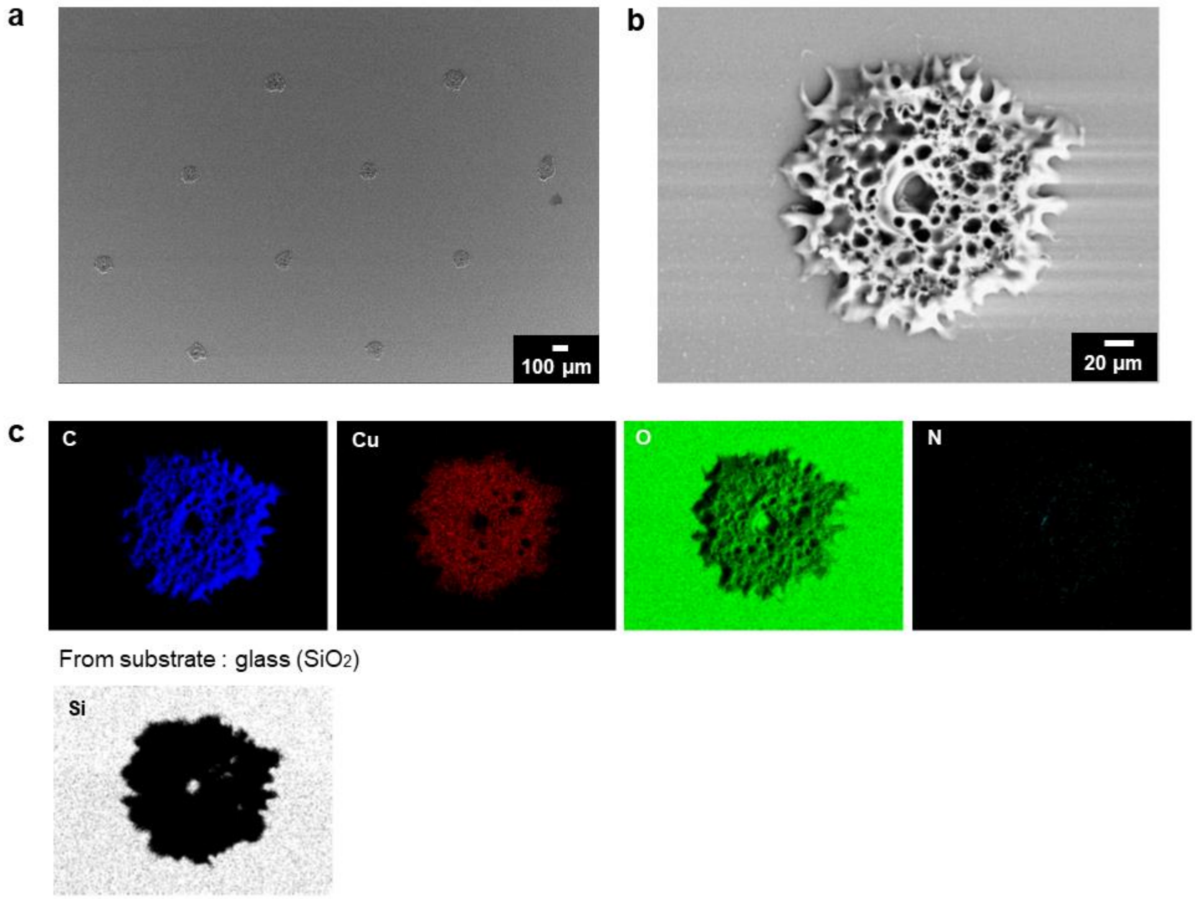


**Supplementary Fig. 16** (a, b) SEM image and (c) EDX mapping of a spot array on a glass substrate, generated with a PVP and copper nitrate donor slide.

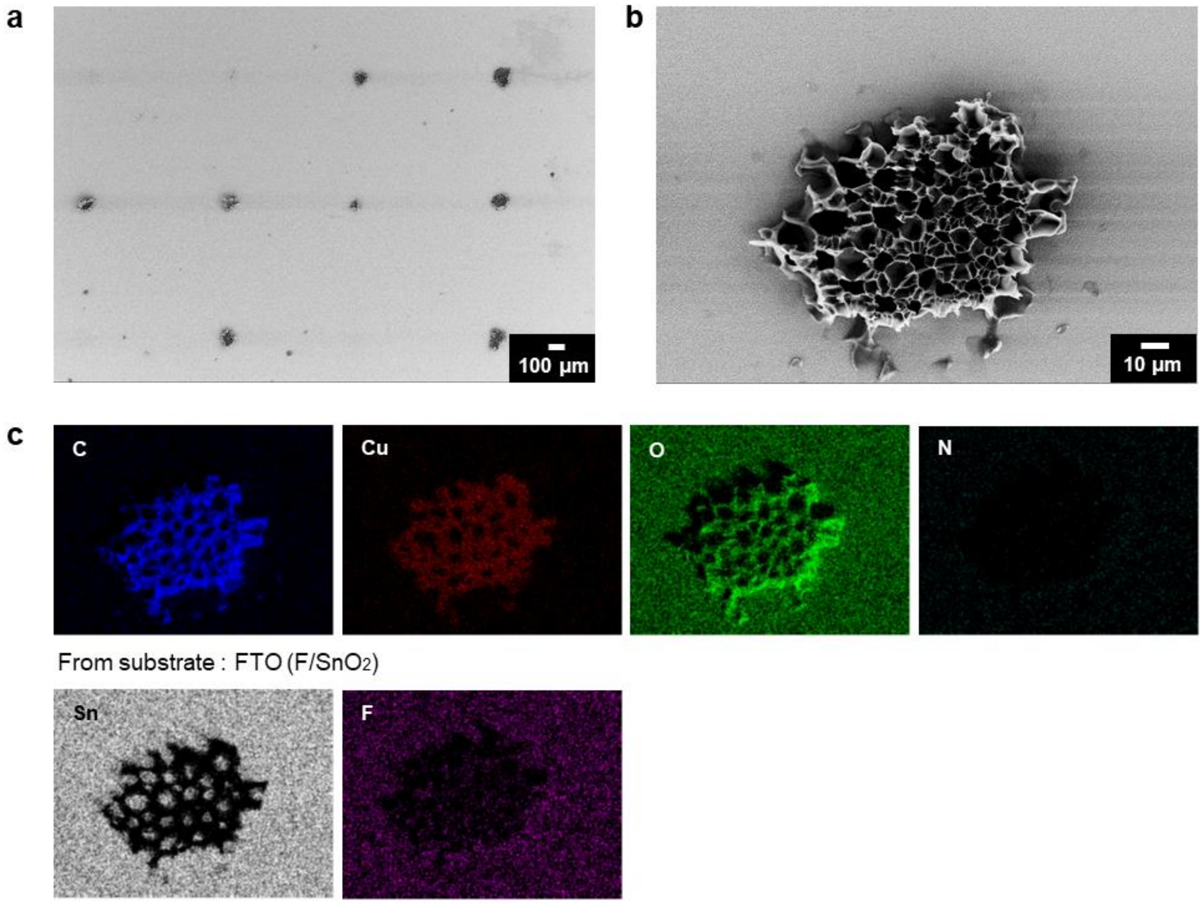


**Supplementary Fig. 17** (a, b) SEM image and (c) EDX mapping of a spot array on an FTO substrate generated with a PVP and copper nitrate donor slide.

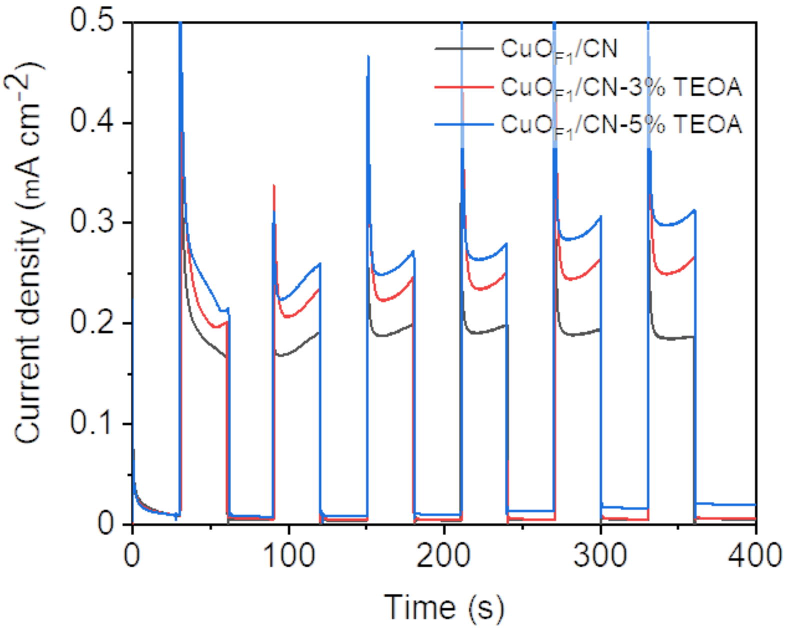


**Supplementary Fig. 18** Transient photocurrent response of $CuO_{F1}$/CN electrodes with and without hole scavenger (TEOA) in 0.1 M NaOH solution at 1.23 V *vs.* RHE (reversible hydrogen electrode).

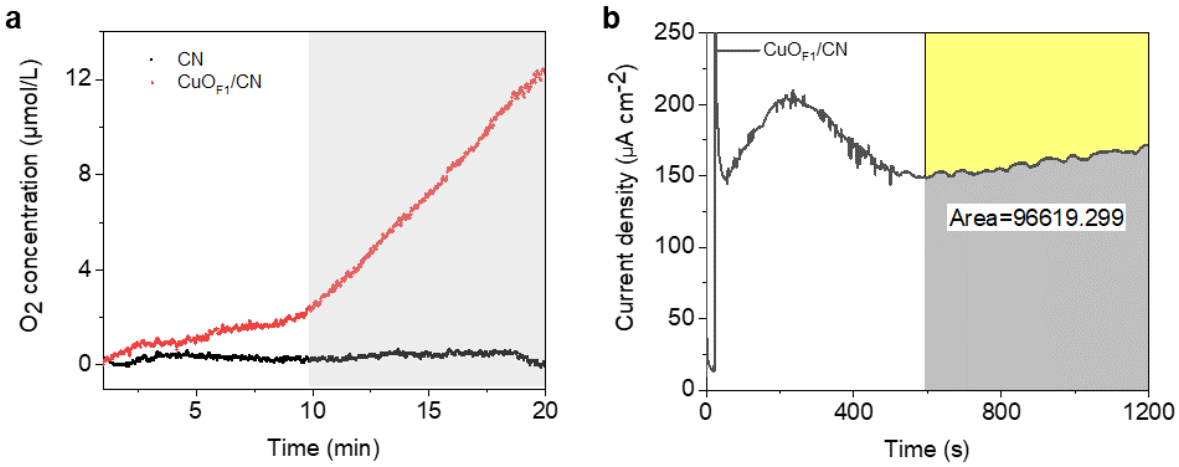


**Supplementary Fig. 19** (a) Oxygen concentration in the electrolyte (baseline due to leakage of oxygen into the electrochemical cell from the environment has been subtracted). (b) Photocurrent density of the composite $CuO_{F1}$/CN electrode and the integration of current with respect to time in the linear region.

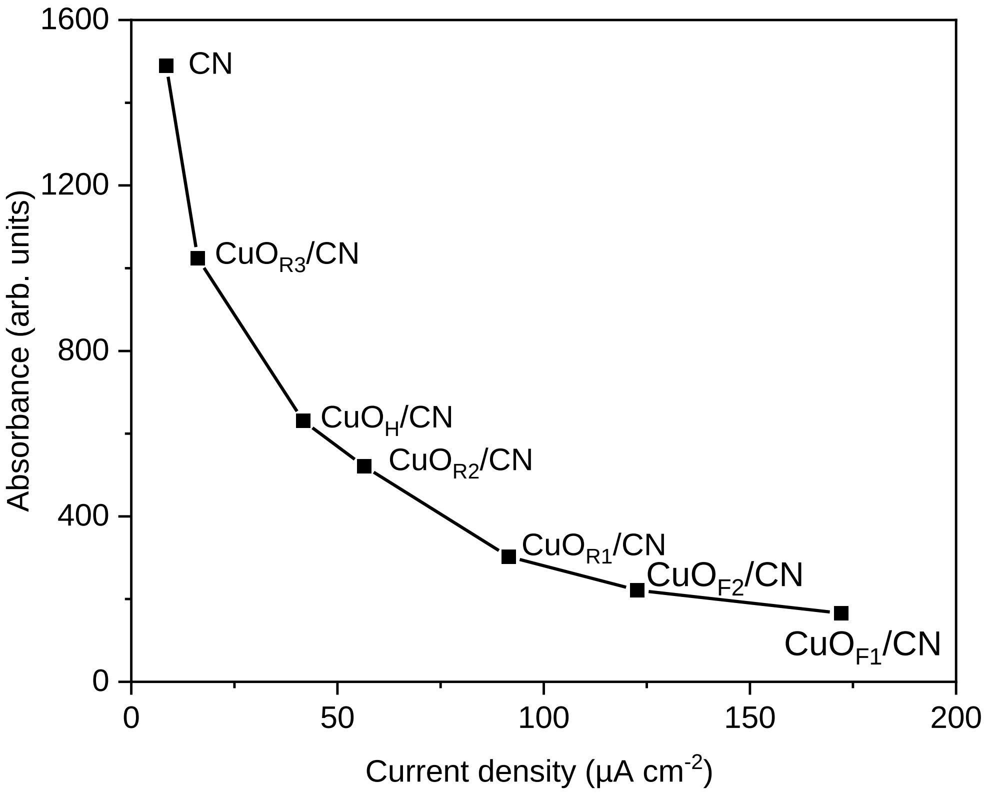


**Supplementary Fig. 20** Increase of photocurrent density in comparison with the steady-state fluorescence intensity. Samples are excited at 365 nm, with an emission peak around 437 nm. The potential was set to 1.23 V vs. RHE. The magnitude of the photocurrent correlates excellently with the results of steady-state fluorescence measurements – the lower fluorescence intensity, the higher the photocurrent.

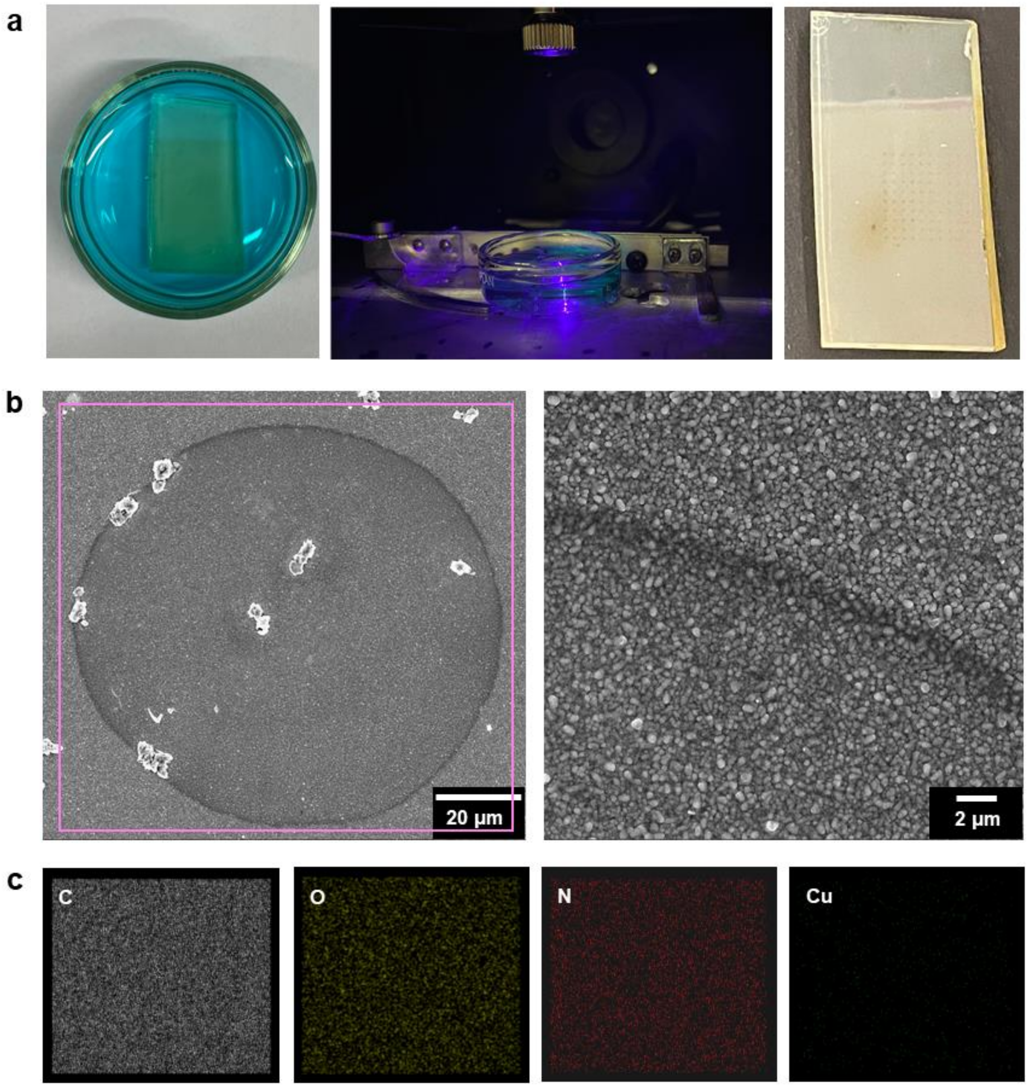


**Supplementary Fig. 21** (a) Photograph of synthesis process: CN substrate in $Cu(NO_3)_2$ solution, laser irradiation, CN with spots array. (b) SEM image and (c) EDX mapping of spot generated by laser irradiation.

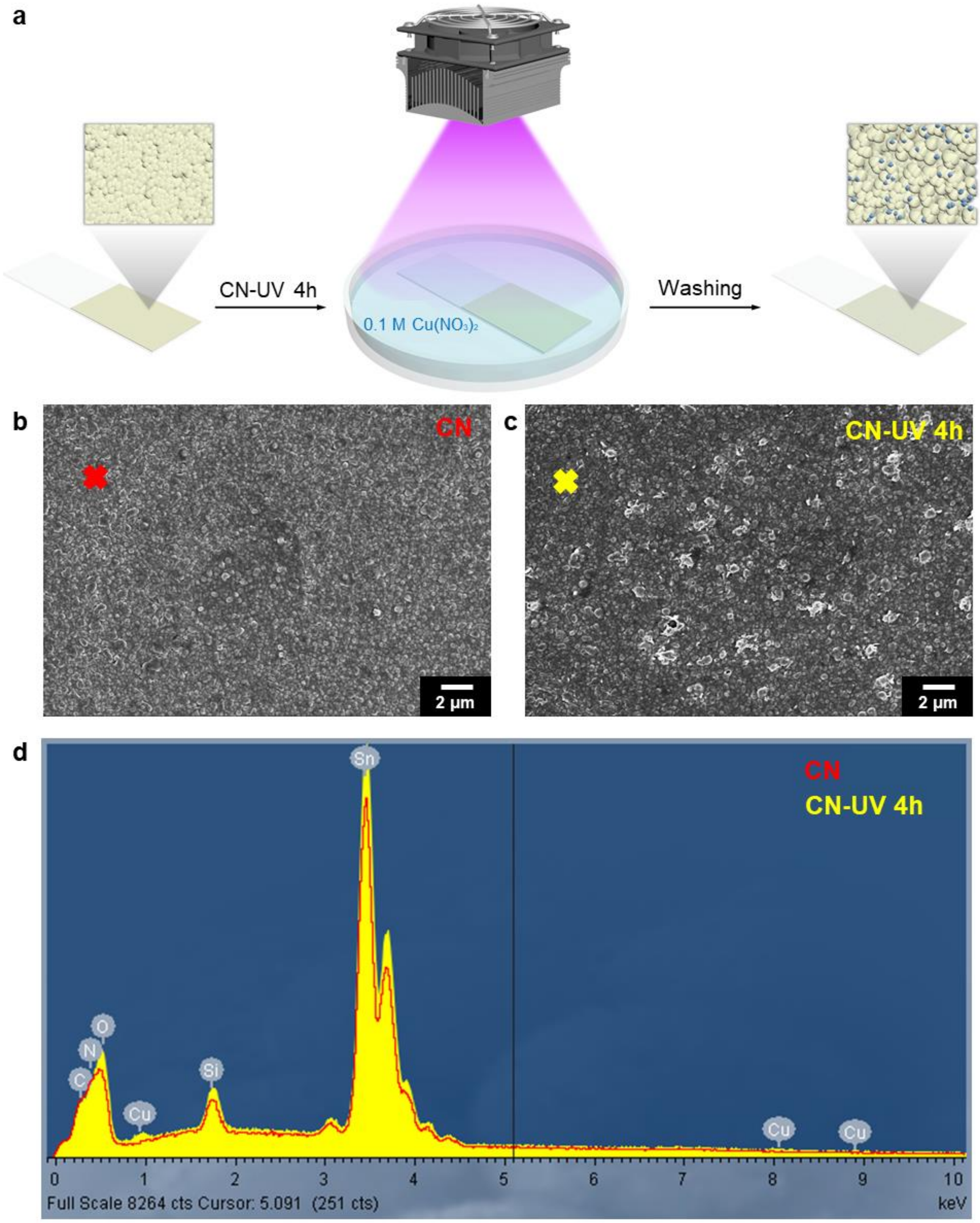


**Supplementary Fig. 22** (a) Schematic representation of the photodeposition process on CN substrate, which in turn was obtained by chemical vapor deposition. SEM image of CN substrate before (b) and after (c) UV irradiation. (d) EDX spectrum of CN substrate before (red) and after (yellow) UV irradiation.

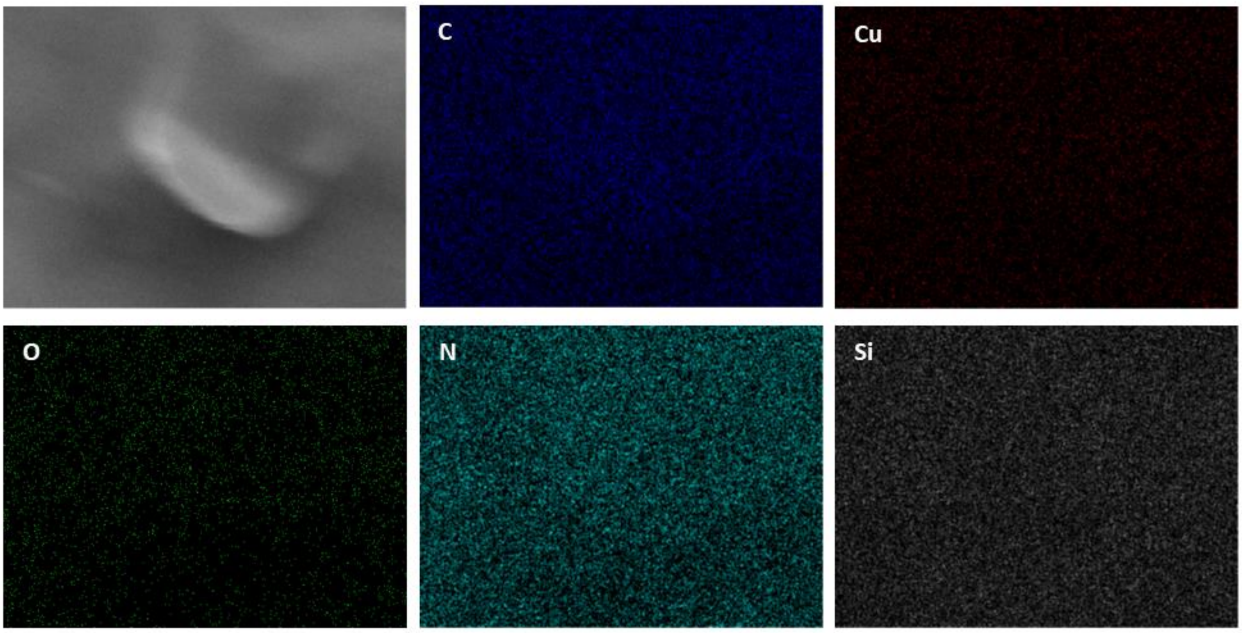


**Supplementary Fig. 23** EDX mapping of rod-like structure on the surface of carbon nitride after 4 h of irradiation with UV.

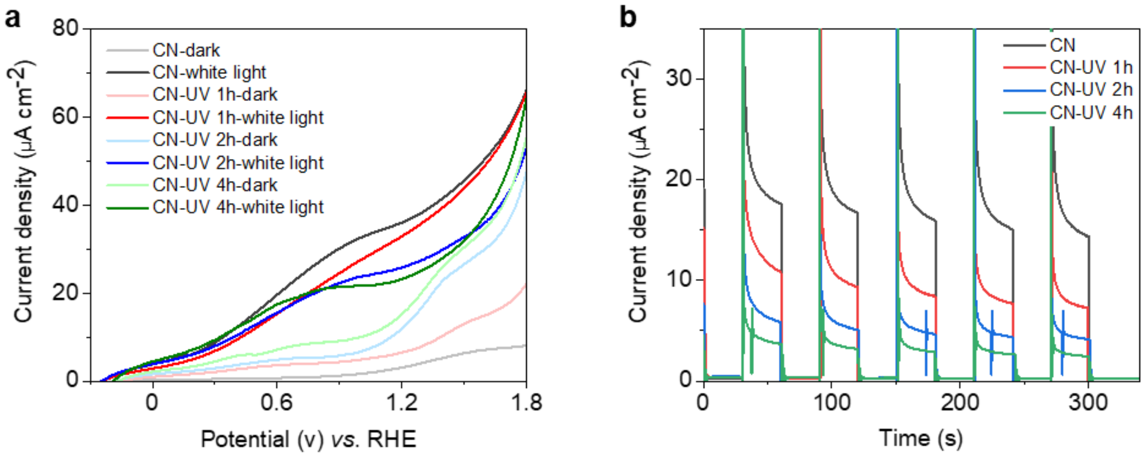


**Supplementary Fig. 24** Photoelectrochemical properties of copper-doped carbon nitride obtained by photodeposition. (a) Linear sweep voltammetry curves (b) chronoamperometry at 1.23 V *vs.* RHE (reversible hydrogen electrode) with or without white light.

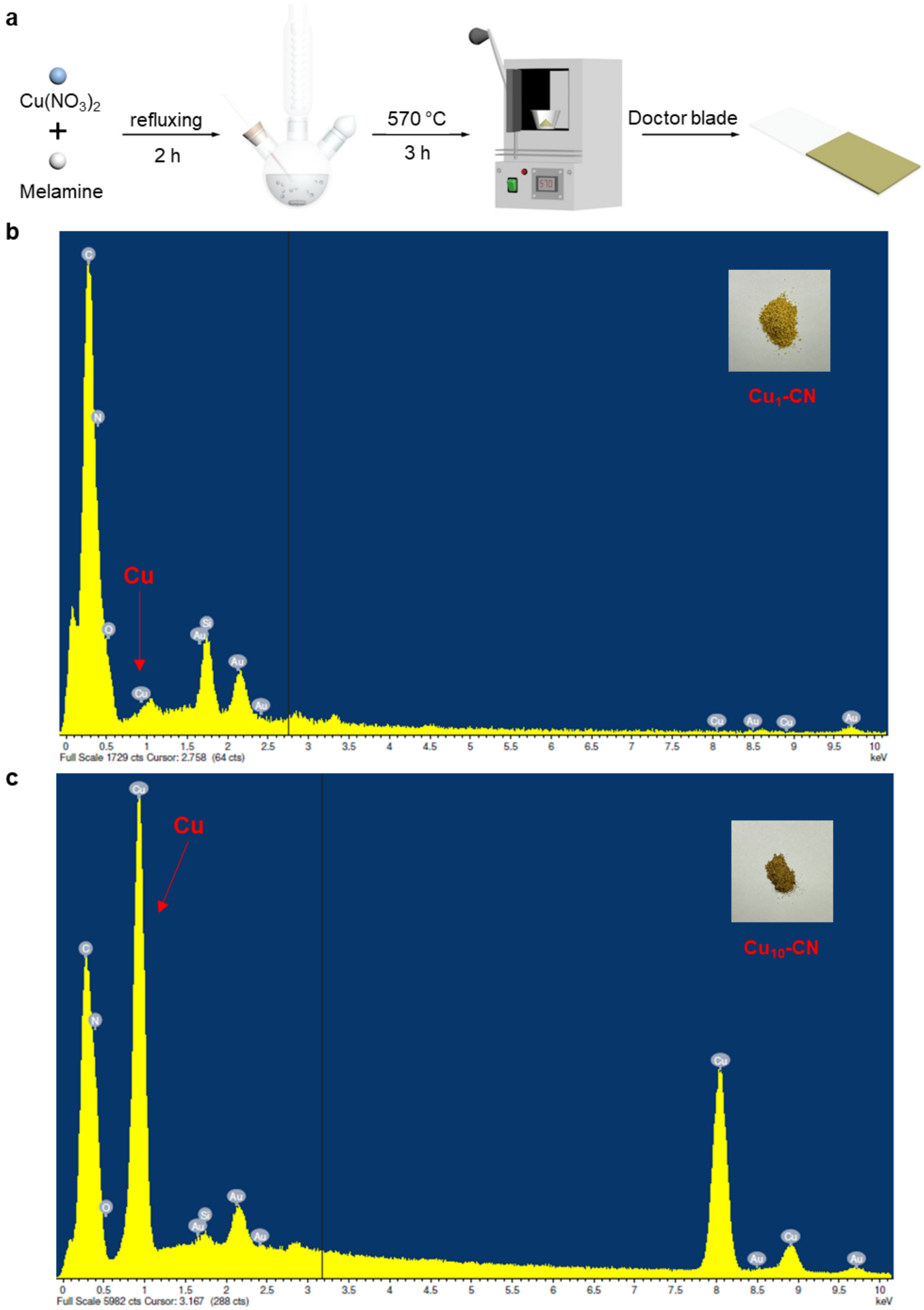


**Supplementary Fig. 25** (a) Schematic representation of copper-doped CN synthesis process by co-polymerization. EDX spectrum of $Cu_1$-CN (b) and $Cu_{10}$-CN (c). Red arrows show the L-alpha (Lα) line of copper.

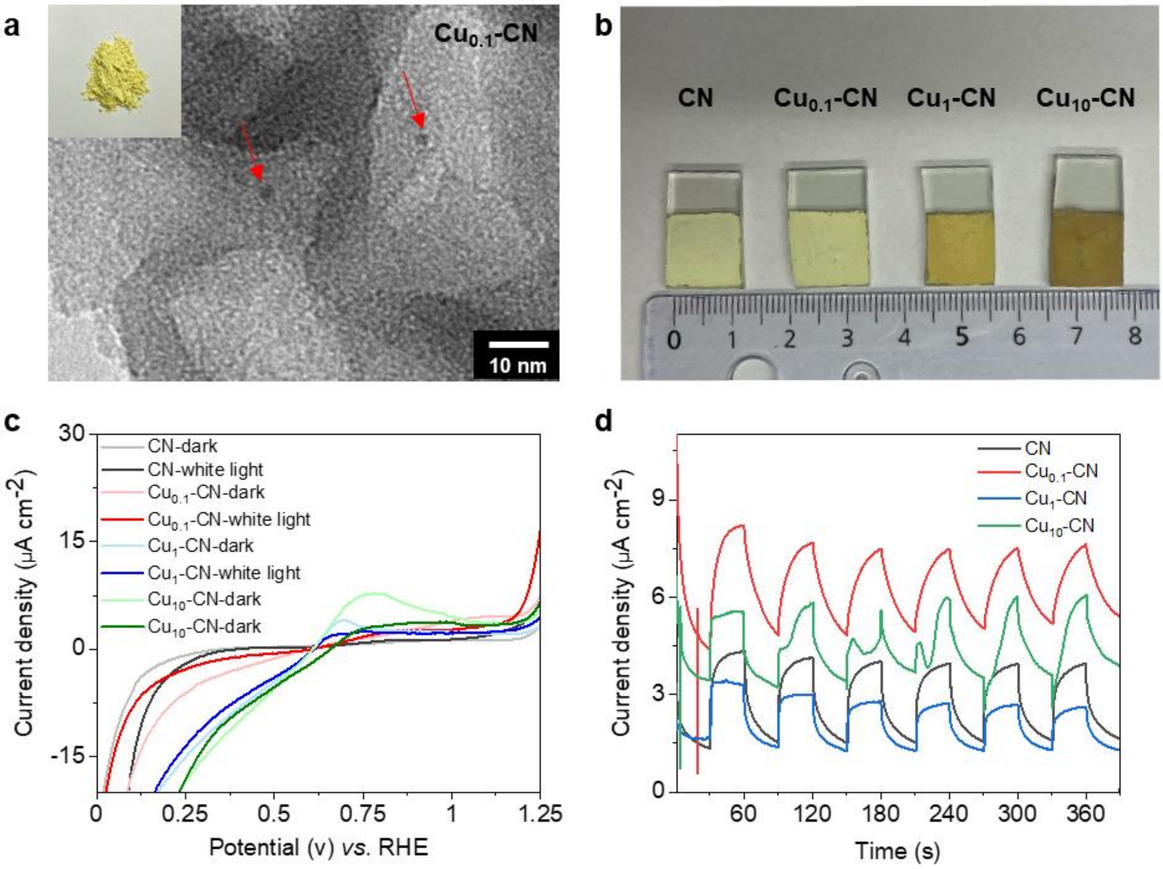


**Supplementary Fig. 26** (a) Photograph and TEM image of $Cu_{0.1}$-CN. (b) Photograph of the electrodes prepared using doctor blade technique. (c) Linear sweep voltammetry curves of the electrodes. (d) Chronoamperometry of electrodes at 1.23 V *vs.* RHE (reversible hydrogen electrode) upon irradiation with white light and in dark.

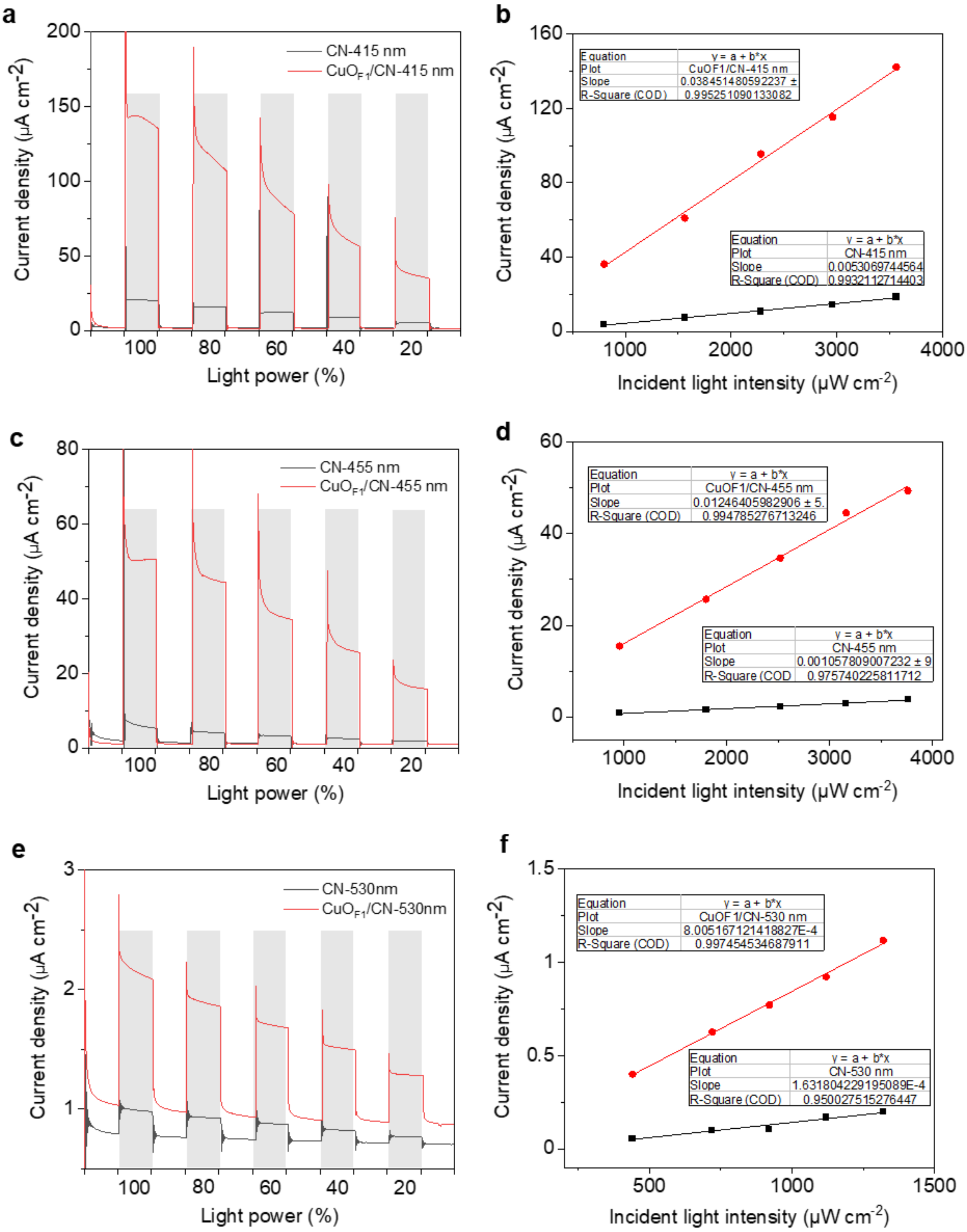
a
b
c
d
e
f
CN-415 nm
CuO$_{F1}$/CN-415 nm
CN-455 nm
CuO$_{F1}$/CN-455 nm
CN-530nm
CuO$_{F1}$/CN-530nm
Current density (μA cm$^{-2}$)
Light power (%)
Incident light intensity (μW cm$^{-2}$)
Equation y = a + b*x
Plot CuOF1/CN-415 nm
Slope 0.038451480592237 ±
R-Square (COD) 0.995251090133082
Equation y = a + b*x
Plot CN-415 nm
Slope 0.0053069744564
R-Square (COD) 0.9932112714403
Equation y = a + b*x
Plot CuOF1/CN-455 nm
Slope 0.01246405982906 ± 5.
R-Square (COD) 0.994785276713246
Equation y = a + b*x
Plot CN-455 nm
Slope 0.001057809007232 ± 9
R-Square (COD 0.975740225811712
Equation y = a + b*x
Plot CuOF 1/CN-530 nm
Slope 8.005167121418827E-4
R-Square (COD) 0.997454534687911
Equation y = a + b*x
Plot CN-530 nm
Slope 1.631804229195089E-4
R-Square (COD) 0.950027515276447

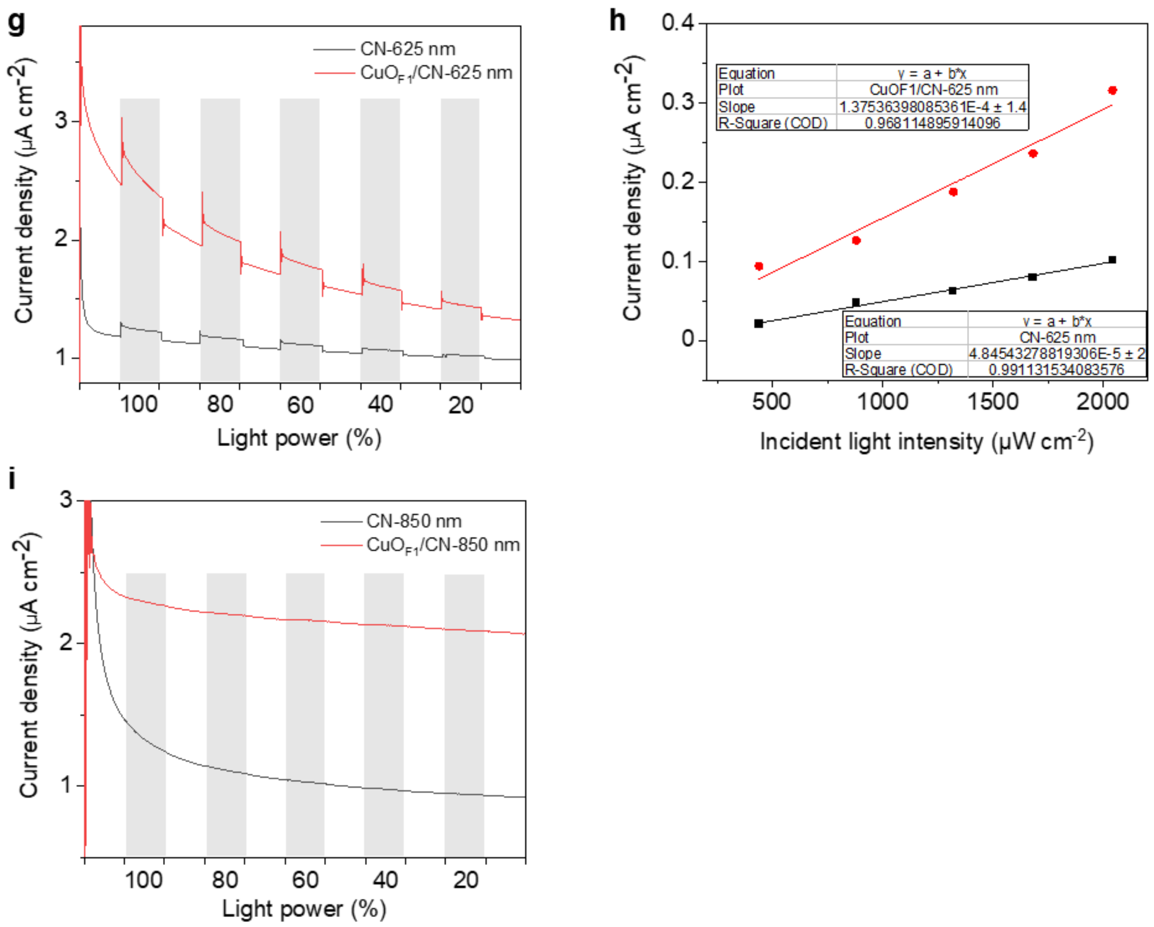


**Supplementary Fig. 27** Photocurrent density of pristine CN and composite $CuO_{F1}$/CN electrodes under irradiation with different light intensity. The corresponding linear fittings between current densities and incident light intensities are shown on the right side. The measurements were performed at 1.23 V *vs*. RHE in 0.1 M NaOH.

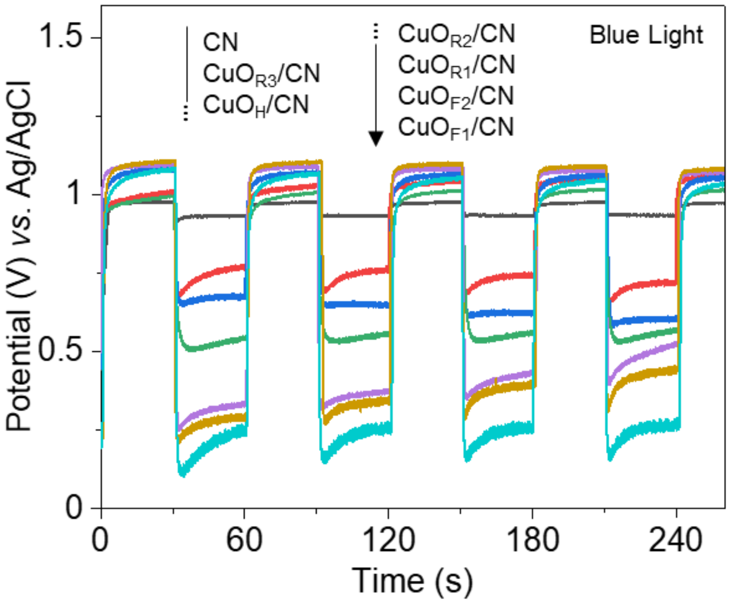


**Supplementary Fig. 28** Chronopotentiometry of $CuO_{F1}$/CN electrodes under 50 μA constant current with blue light irradiation.

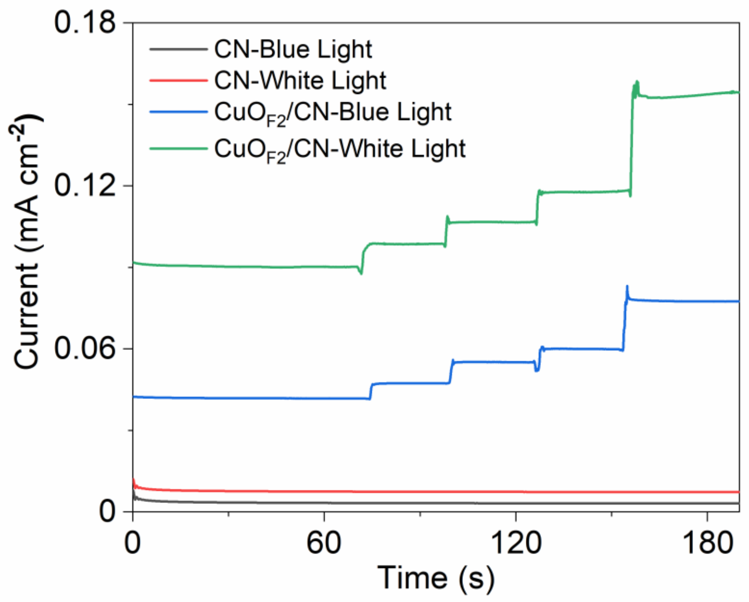


**Supplementary Fig. 29** Photocurrent response of CuO/CN and CN electrodes to glucose injection under white light or blue light in 0.1 M NaOH solution.

The current response is measured during the successive injection of glucose. $CuO_{f2}$/CN photoelectrode in 0.1 M NaOH electrolyte solution is chosen for the detection. A fast response is observed at 0.1 V (*vs.* Ag/AgCl reference electrode) with both white and blue light, which is a 3 – 7 times lower potential than other reported CuO-based composite films. This is likely achieved by the input of incident light and the amplifying cooperation of CuO and CN.

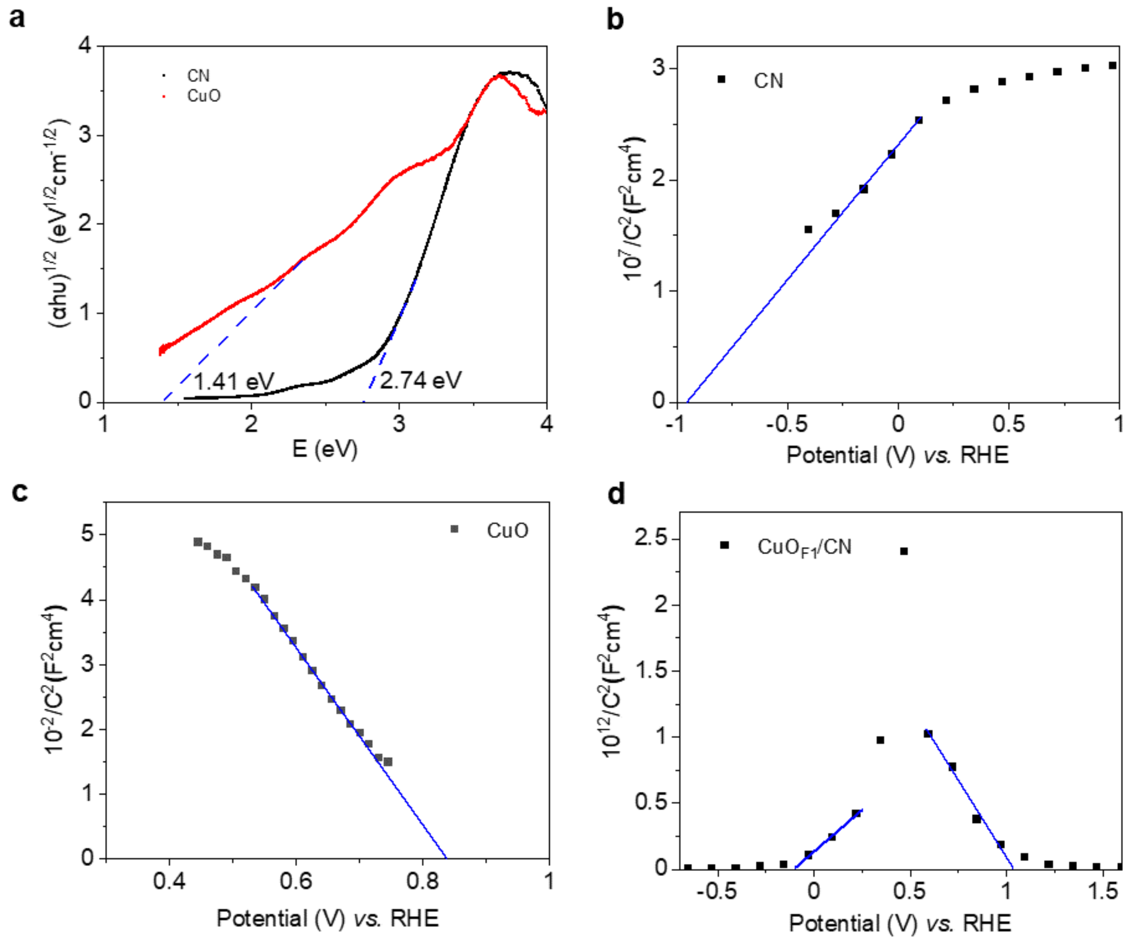


**Supplementary Fig. 30** (a) Tauc plots of CN and CuO. (b) Mott-Schottky plots of CN, (c) CuO, and (d) $CuO_{F1}$/CN electrodes at 15 Hz.

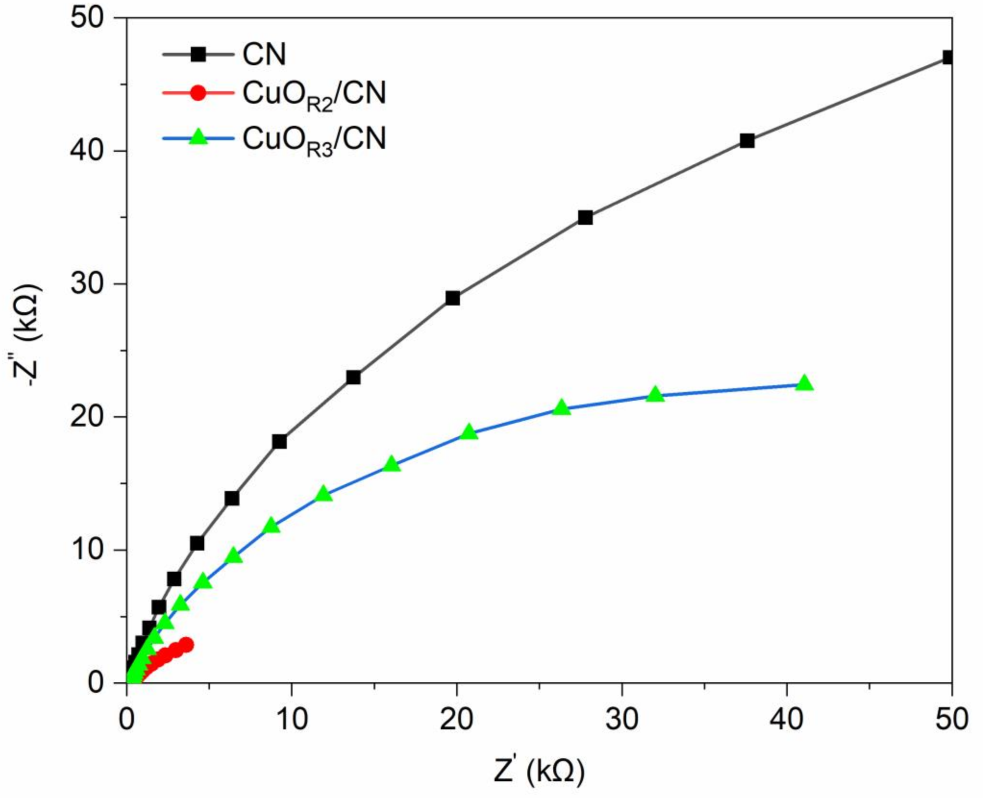


**Supplementary Fig. 31** Nyquist plots for CN, CuO$_{R2}$/CN (single-layer CuO nanorods) and CuO$_{R3}$/CN (multi-layer CuO nanorods).

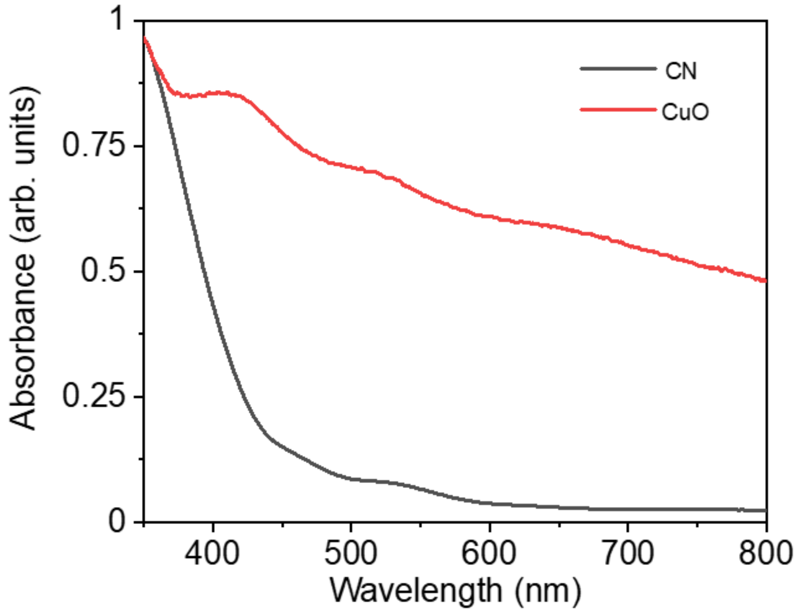


**Supplementary Fig. 32** UV-visible absorption spectra of CN and CuO electrodes.

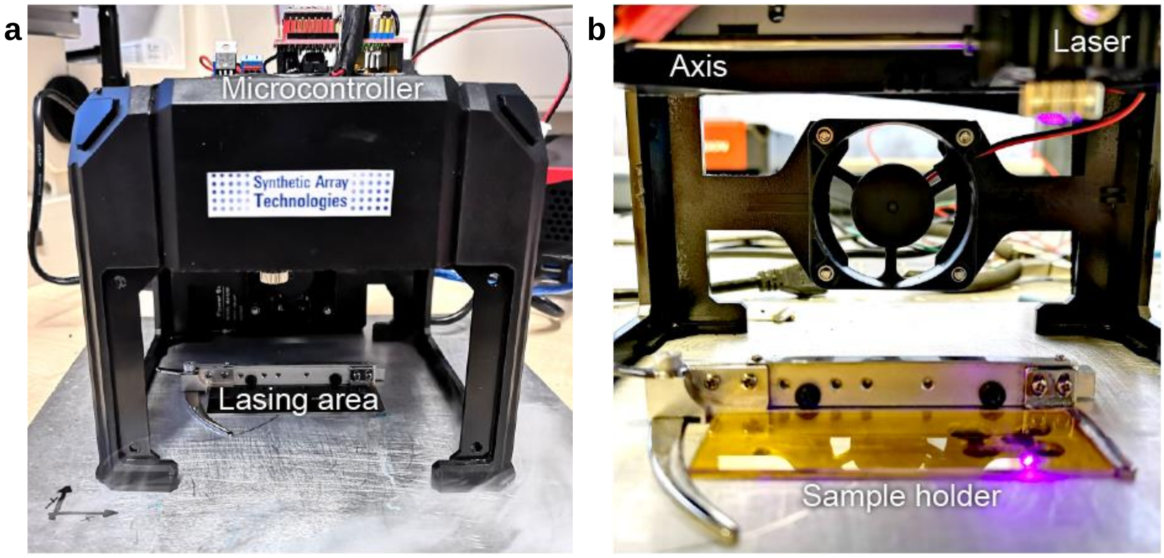


**Supplementary Fig. 33** 405 nm LTRAS machine setup showing the different components.

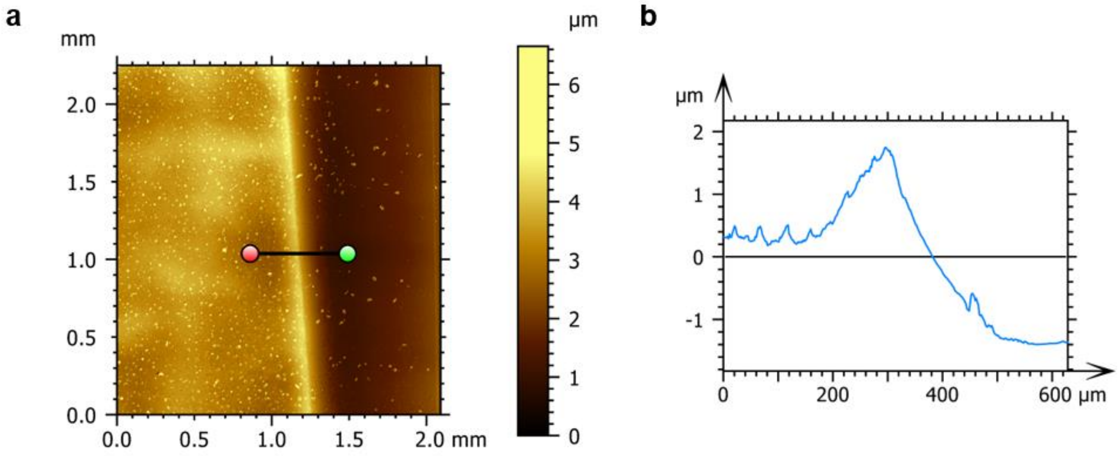


**Supplementary Fig. 34** (a) Vertical scanning interferometry (VSI) measurement of donor slide and (b) thickness profile.

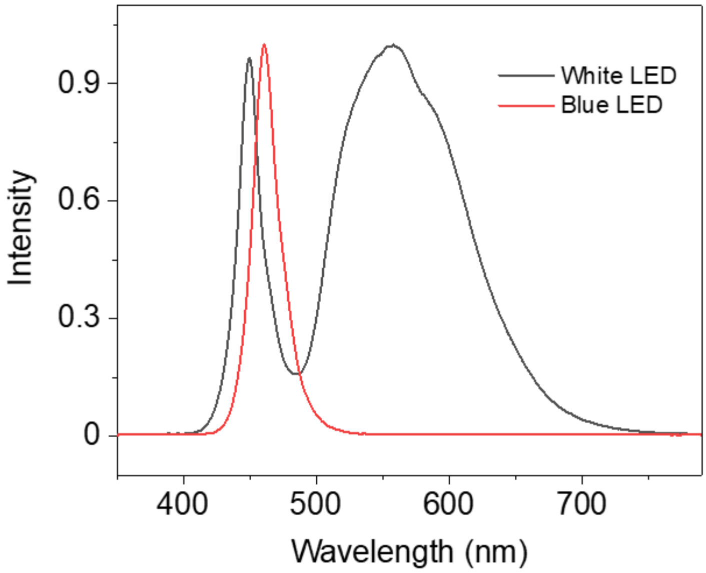


**Supplementary Fig. 35** Measured intensity vs. wavelength spectra of the white (300 mW/cm²) and blue (100 mW/cm²) LED lamps. White light: CHANZON 50 W LED Chip, 6000–6500K color temperature, 30–34V, 1500 mA. Blue light: CHANZON 50 W LED Chip, 460–470 nm, 30–34 V, 1500 mA.

**Supplementary Table 1** Summary of CuO nanostructures obtained by different methods

| **Morphology** | **Method** | **Size** | **Precursor** | **Temperature** | **Duration** |
|---|---|---|---|---|---|
| Nanorods[6] | Hydrothermal | Width: 30−160 nm<br>Length: 0.9–1.6 µm | $Cu(NO_3)_2$ | 140 °C | 12–36 h |
| Nanobats[7] | Hydrothermal | Width: 70 nm<br>Length: 170 nm | $Cu(NO_3)_2$ | 100–150 °C | 6–15 h |
| Hierarchical microflowers[8] | Hydrothermal | 5–10 µm | $CuSO_4$ | 120 °C | 6 h |
| Spindle-like structure[9] | Chemical precipitation | Width: 130 nm<br>Length: 320 nm | $Cu(NO_3)_2$<br>NaOH | 80 °C | 30 min |
| Pillow-like structure[10] | Solid-state thermal conversion | 1.5 µm | $CuSO_4$ | 350 °C | 4 h |
| Nanowires[11] | Thermal oxidation | Diameter: 30–200 nm<br>Length: up to 15 µm | Cu | 500 °C | 4 h |
| Microflowers and nanospindles[12] | Thermal decomposition | 10 µm<br>60 nm | $CuSO_4$<br>$Cu(NO_3)_2$ | 250 °C | 3 h |
| Spherical flower-like structure[13] | Microwave-assisted hydrothermal | 0.7–2 µm | $Cu(CH_3COO)_2$<br>$Cu(NO_3)_2$ | 120–150 °C | 30 min |
| Nanorods and flower-like structure (**this work**) | Laser-driven transfer synthesis | Diameter: 30–50 nm<br>Length: 200–450 nm | $Cu(NO_3)_2$ | 500 °C | 0.02–0.04 s |

**Supplementary Table 2** PEC performances of recent publications involving CN photoanodes.

| **CN electrodes** | **Photocurrent ($\mu A\ cm^{-2}$)** | **Operation Potential** | **Electrolyte** | **Light intensity** |
|---|---|---|---|---|
| CN-rGO[14] | 75 | 1.23 V *vs*. RHE | 0.1 M KOH | 1.5 AM |
| B-doped CN[15] | 103 | 1.23 V *vs*. RHE | 0.1 M $Na_2SO_4$ | 1.5 AM |
| S-doped CN[16] | 100 | 1.23 V *vs*. RHE | 1.0 M NaOH | 1.5 AM |
| CN/B-doped CN/P-doped CN composites[17] | 150 | 1.23 V *vs*. RHE | 0.1 M $Na_2SO_4$ | 1.5 AM |
| $TiO_2$/CN[18] | 110 | 1.23 V *vs*. RHE | 0.1 M KCl | Xe lamp, 100 mW $cm^{-2}$ |
| 3%Ni-CN[19] | 75.6 | 1.23 V *vs*. RHE | 0.1 M KOH | 1.5 AM |
| CuO/CN [20] | 11 | −0.1 V vs. SCE | 0.1 M PBS | Xe lamp, 100 mW $cm^{-2}$ |
| CuO/CN (**this work**) | 172 | 1.23 V *vs*. RHE | 0.1 M NaOH | White light 511 mW $cm^{-2}$ |
| CuO/CN (**this work**) | 225 | 1.23 V *vs*. RHE | 0.1 M NaOH + 5 % TEOA | White light 511 mW $cm^{-2}$ |
| CuO/CN (**this work**) | 65.4 | 1.23 V *vs*. RHE | 0.1 M NaOH | Blue light 101 mW $cm^{-2}$ |

**Supplementary Table 3** List of copper based non-enzymatic glucose sensors

| No. | Electrodes | Operation Potential | Medium | Response time (s) |
|---|---|---|---|---|
| **1** | CuO nanowires [21] | +0.55 V *vs.* Ag/AgCl | 0.05 M NaOH | <5 s |
| **2** | $Cu^{2+}$ doped CN/MWCNT[22] | +0.6 V *vs.* SCE | 0.1 M NaOH | ~1 s |
| **3** | CuO/rGO[23] | +0.55 V *vs.* Ag/AgCl | 0.1 M NaOH | 6 |
| **4** | CuO/carbon fiber fabric[24] | +0.45 V *vs.* Ag/AgCl | 0.1 M NaOH | 1.3 |
| **5** | SiO2/C/CuO[25] | +0.6 V *vs.* SCE | 0.1 M NaOH | ~1 s |
| **6** | PDDA/CuO-C-dot[26] | +0.5 V *vs.* Ag/AgCl | 0.1 M NaOH | N/A |
| **7** | CuO/Cu/$TiO_2$[27] | +0.65 V *vs.* Ag/AgCl | 0.1 M NaOH | ~1 s |
| **8** | PbS/$SiO_2$/AuNPs[28] | -0.2 V *vs.* SCE | 0.1 M Tris–HCl buffer | ~1 s |
| **9** | Graphene/$WO_3$/Au[29] | -0.4 V vs.Ag/AgCl | 0.1 M PBS buffer | ~1 s |
| **10** | GO/CdS[30] | +0.2 V *vs.* SCE | 0.1 M NaOH | Not available |
| **11** | $BiVO_4$[31] | +0.15 V *vs.* Ag/AgCl | 0.1 M $NaNO_3$ | ~1 s |
| **12** | CoOx/graphene-CdS[32] | +0.4 V *vs.* Ag/AgCl | 0.1 M NaOH | ~3 s |
| **13** | CuO/$TiO_2$/SPCE[33] | +0.7 V *vs.* Ag/AgCl | 0.1 M KOH | Not available |
| **14** | CuO-ZnO[34] | +0.62 V *vs.* Ag/AgCl | 0.1 M NaOH | <2 s |
| **15** | CuO/CN (this work) | +0.1 V *vs.* Ag/AgCl | 0.1 M NaOH | ~1 s |

**Supplementary Table 4** Effective absorbed laser energy fluences for the two laser setups at different laser powers

| Laser | $d_{spot}$ (1/e$^2$) | Power | Speed | $t_{EIR}$ | Effective (absorbed) fluence |
|---|---|---|---|---|---|
| **405 nm** | 42 µm | 42 mW | 16.7 µm/ms | 2.51 ms | 28.0 J/cm$^2$ |
| **405 nm** | 42 µm | 25 mW | 16.7 µm/ms | 2.51 ms | 16.7 J/cm$^2$ |
| **488 nm** | 18 µm | 90 mW | 35 µm/ms | 0.51 ms | 8.1 J/cm$^2$ |
| **488 nm** | 18 µm | 76,4 mW | 35 µm/ms | 0.51 ms | 6.8 J/cm$^2$ |
| **488 nm** | 18 µm | 90 mW | 60 µm/ms | 0.30 ms | 4.7 J/cm$^2$ |
| **488 nm** | 18 µm | 76,4 mW | 60 µm/ms | 0.30 ms | 4.0 J/cm$^2$ |

## References


[1] H. Riegler, R. Köhler, *Nature Physics* **2007**, *3*, 890-894.

[2] J. Zhang, S. Gim, G. Paris, P. Dallabernardina, C. N. Z. Schmitt, S. Eickelmann, F. F. Loeffler, *Chem. - Eur. J.* **2020**, *26*, 1243-1248.

[3] B. Liu, H. C. Zeng, *J. Am. Chem. Soc.* **2004**, *126*, 8124-8125.

[4] aQ. Zhang, S. J. Liu, S. H. Yu, *J. Mater. Chem.* **2009**, *19*, 191-207; bZ. Zhang, H. Sun, X. Shao, D. Li, H. Yu, M. Han, *Adv. Mater.* **2005**, *17*, 42-47.

[5] aW. Zhang, X. Wen, S. Yang, Y. Berta, Z. L. Wang, *Adv. Mater.* **2003**, *15*, 822-825; bQ. Zhang, K. Zhang, D. Xu, G. Yang, H. Huang, F. Nie, C. Liu, S. Yang, *Prog. Mater. Sci.* **2014**, *60*, 208-337.

[6] K. M. Shrestha, C. M. Sorensen, K. J. Klabunde, *J. Phys. Chem. C* **2010**, *114*, 14368-14376.

[7] M. A. Dar, Q. Ahsanulhaq, Y. S. Kim, J. M. Sohn, W. B. Kim, H. S. Shin, *Appl. Surf. Sci.* **2009**, *255*, 6279-6284.

[8] Z. Cheng, J. Xu, H. Zhong, X. Chu, J. Song, *Mater. Lett.* **2011**, *65*, 2047-2050.

[9] S. Sun, X. Zhang, J. Zhang, L. Wang, X. Song, Z. Yang, *CrystEngComm* **2013**, *15*, 867-877.

[10] M. Wan, D. Jin, R. Feng, L. Si, M. Gao, L. Yue, *Inorg. Chem. Commun.* **2011**, *14*, 38-41.

[11] X. Jiang, T. Herricks, Y. Xia, *Nano Lett.* **2002**, *2*, 1333-1338.

[12] M. Chawla, V. Sharma, J. K. Randhawa, *Electrocatalysis* **2017**, *8*, 27-35.

[13] G. Qiu, S. Dharmarathna, Y. Zhang, N. Opembe, H. Huang, S. L. Suib, *J. Phys. Chem. C* **2012**, *116*, 468-477.

[14] G. Peng, M. Volokh, J. Tzadikov, J. Sun, M. Shalom, *Adv. Energy Mater.* **2018**, *8*, 1800566.

[15] Q. Ruan, W. Luo, J. Xie, Y. Wang, X. Liu, Z. Bai, C. J. Carmalt, J. Tang, *Angew. Chem., Int. Ed.* **2017**, *56*, 8221-8225.

[16] Y. Fang, X. Li, X. Wang, *ACS Catal.* **2018**, *8*, 8774-8780.

[17] P. Luan, Q. Meng, J. Wu, Q. Li, X. Zhang, Y. Zhang, L. A. O'Dell, S. R. Raga, J. Pringle, J. C. Griffith, C. Sun, U. Bach, J. Zhang, *ChemSusChem* **2020**, *13*, 328-333.

[18] T. Zhao, Q. Zhou, Y. Lv, D. Han, K. Wu, L. Zhao, Y. Shen, S. Liu, Y. Zhang, *Angew. Chem., Int. Ed.* **2020**, *59*, 1139-1143.

[19] W. Zhang, J. Albero, L. Xi, K. M. Lange, H. Garcia, X. Wang, M. Shalom, *ACS Appl. Mater. Interfaces* **2017**, *9*, 32667-32677.

[20] L. Yang, Z. Zhao, J. Hu, H. Wang, J. Dong, X. Wan, Z. Cai, M. Li, *Electroanalysis* **2020**, *32*, 1651-1658.

[21] Y. Zhang, Y. Liu, L. Su, Z. Zhang, D. Huo, C. Hou, Y. Lei, *Sens. Actuators, B* **2014**, *191*, 86-93.

[22] W. Zheng, Y. Li, M. Liu, C.-S. Tsang, L. Y. S. Lee, K.-Y. Wong, *Electroanalysis* **2018**, *30*, 1446-1454.

[23] Y. Zhao, X. Bo, L. Guo, *Electrochim. Acta* **2015**, *176*, 1272-1279.

[24] W. Xu, S. Dai, X. Wang, X. He, M. Wang, Y. Xi, C. Hu, *J. Mater. Chem. B* **2015**, *3*, 5777-5785.

[25] A. Rahim, Z. U. Rehman, S. Mir, N. Muhammad, F. Rehman, M. H. Nawaz, M. Yaqub, S. A. Siddiqi, A. A. Chaudhry, *J. Mol. Liq.* **2017**, *248*, 425-431.

[26] T. U. Sridara, J.; Saianand, G.; Tuantranont, A.; Karuwan, C.; Jakmunee, J., *Sensors* **2020**, *20*, 808.

[27] Z. Zhou, Z. Zhu, F. Cui, J. Shao, H. S. Zhou, *Microchim. Acta* **2020**, *187*, 123.

[28] L. Cao, P. Wang, L. Chen, Y. Wu, J. Di, *RSC Advances* **2019**, *9*, 15307-15313.

[29] A. Devadoss, P. Sudhagar, S. Das, S. Y. Lee, C. Terashima, K. Nakata, A. Fujishima, W. Choi, Y. S. Kang, U. Paik, *ACS Applied Materials & Interfaces* **2014**, *6*, 4864-4871.
[30] X. Zhang, F. Xu, B. Zhao, X. Ji, Y. Yao, D. Wu, Z. Gao, K. Jiang, *Electrochimica Acta* **2014**, *133*, 615-622.
[31] S. Wang, S. Li, W. Wang, M. Zhao, J. Liu, H. Feng, Y. Chen, Q. Gu, Y. Du, W. Hao, *Sensors and Actuators B: Chemical* **2019**, *291*, 34-41.
[32] S. L. Zhimin Ma, *Int. J. Electrochem. Sci.* **2019**, *14*, 11445–11455.
[33] D. M. Tobaldi, C. Espro, S. G. Leonardi, L. Lajaunie, M. P. Seabra, J. J. Calvino, S. Marini, J. A. Labrincha, G. Neri, *Journal of Materials Chemistry C* **2020**, *8*, 9529-9539.
[34] R. Ahmad, N. Tripathy, M.-S. Ahn, K. S. Bhat, T. Mahmoudi, Y. Wang, J.-Y. Yoo, D.-W. Kwon, H.-Y. Yang, Y.-B. Hahn, *Scientific Reports* **2017**, *7*, 5715.
